# Supplementary material for: Synthesis, Spectroscopic Characterization, Antibacterial Activity, and Computational Studies of Novel Pyridazinone Derivatives
Source: Molecules. 2023 Jan 9;28(2):678. doi: 10.3390/molecules28020678 (PMC9861222; doi:10.3390/molecules28020678)
Supplement: Supplementary file 1 [file molecules-28-00678-s001.zip › molecules-2129103-supplementary.pdf]

Supporting Information

Synthesis, Spectroscopic Characterization, Antibacterial Activity, and Computational Studies of Novel Pyridazinone Derivatives

Said Daoui [1], Şahin Direkel [2], Munjed M. Ibrahim [3], Burak Tüzün [4], Tarik Chelfi [1], Mohammed Al-Ghorbani [5,*], Mustapha Bouatia [6], Miloud El Karbane [6], Anass Doukkali [6], Noureddine Benchat [1], Khalid Karrouchi [6,*]

[1] Laboratory of Applied Chemistry and Environment (LCAE), Department of Chemistry, Faculty of Sciences, University Mohammed I, Oujda 60000, Morocco
[2] Department of Medical Microbiology, Faculty of Medicine, Giresun University, Giresun 28100, Turkey
[3] Department of Pharmaceutical Chemistry, College of Pharmacy, Umm Al-Qura University, Makkah 21955, Saudi Arabia; mmsibrahim@uqu.edu.sa
[4] Science Faculty, Department of Chemistry, Cumhuriyet University, Sivas 58140, Turkey
[5] Department of Chemistry, Ulla Science and Art College, Taibah University, KSA, Medina 42353, Saudi Arabia
[6] Laboratory of Analytical Chemistry and Bromatology, Team of Formulation and Quality Control of Health Products, Faculty of Medicine and Pharmacy, Mohammed V University in Rabat, Rabat 10100, Morocco
* Correspondence: mghorbani@taibahu.edu.sa (M.A.-G.); khalid.karrouchi@um5s.net.ma (K.K.)

Table of contents

1. Figure S1. Shapes of optimized structures……………..…………………………...S3-S5

2. Copies of FT-IR spectra……………………………………………………….S6-S11

3. Copies of ^{1}H-NMR and ^{13}C-NMR spectra…..…………………………………….S12-S22

4. Copies of HRMS spectra …..……….……………………………………………...S23-S34

5. Table S1 ……………………………………………………………………...S35-S37

6. Table S2 ……………………………………………………………………...S38-S39

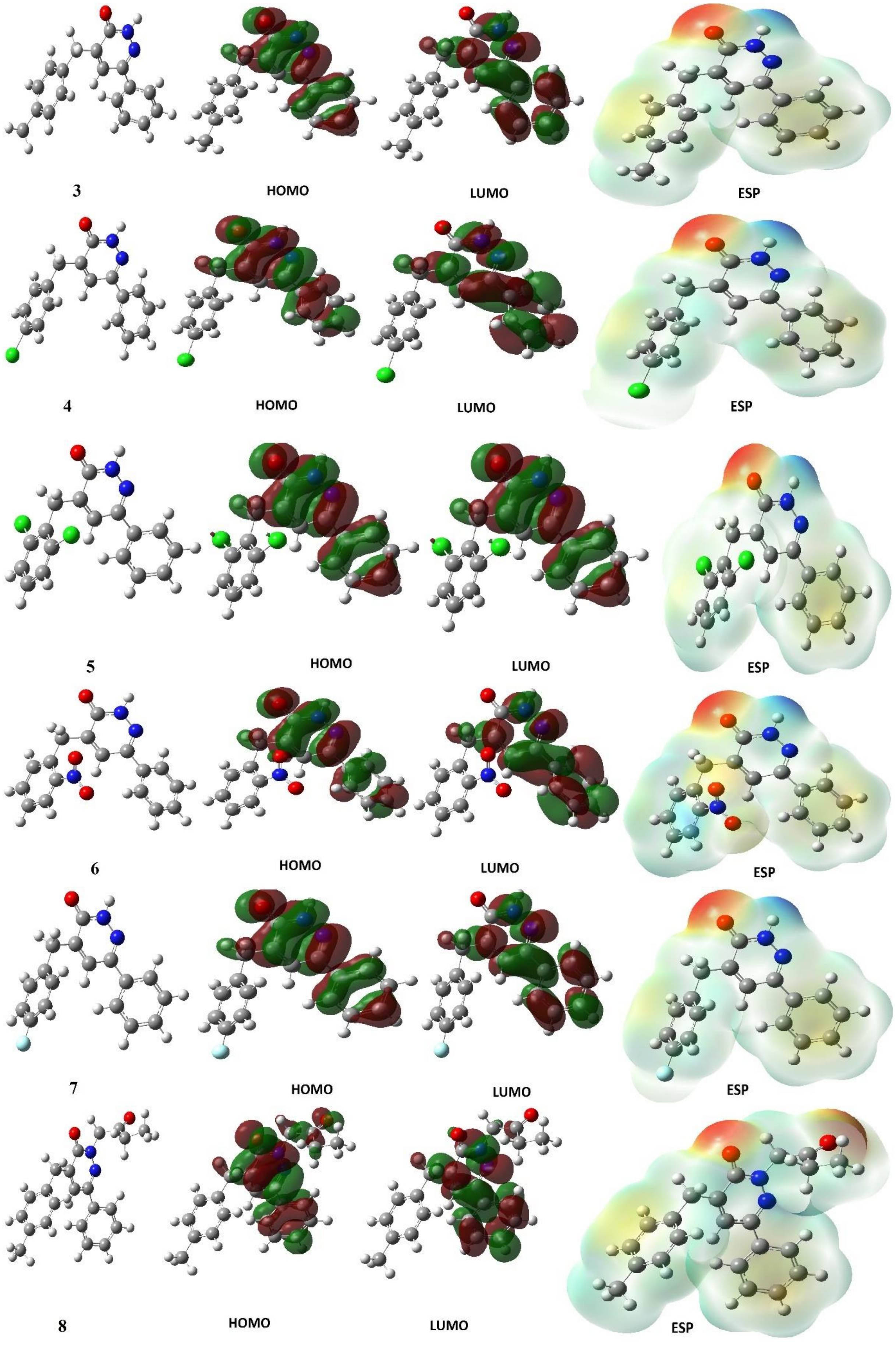

3
HOMO
LUMO
ESP
4
HOMO
LUMO
ESP
5
HOMO
LUMO
ESP
6
HOMO
LUMO
ESP
7
HOMO
LUMO
ESP
8
HOMO
LUMO
ESP

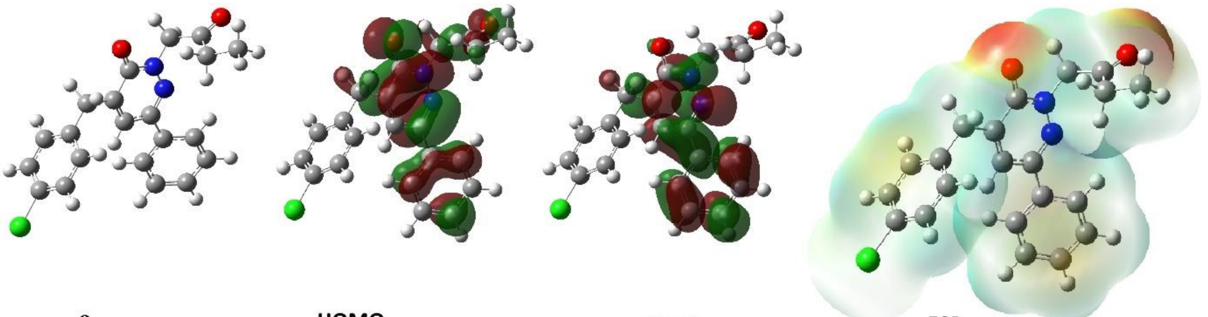

9 HOMO LUMO ESP

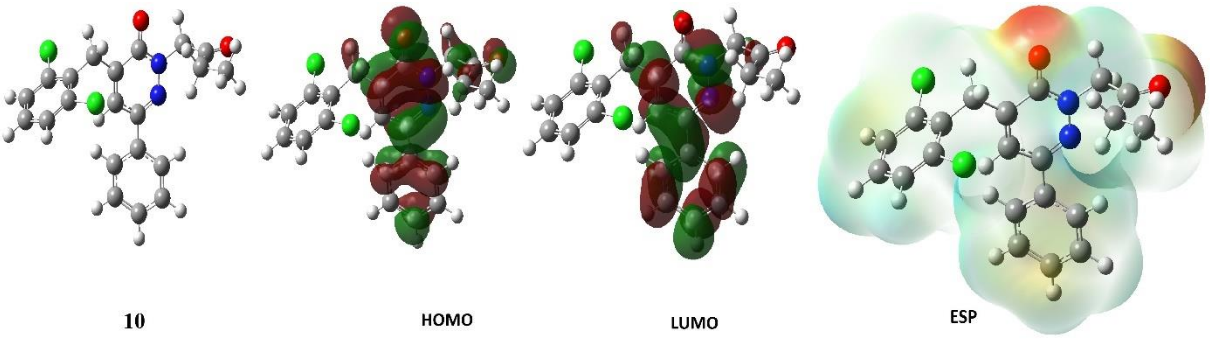

10 HOMO LUMO ESP

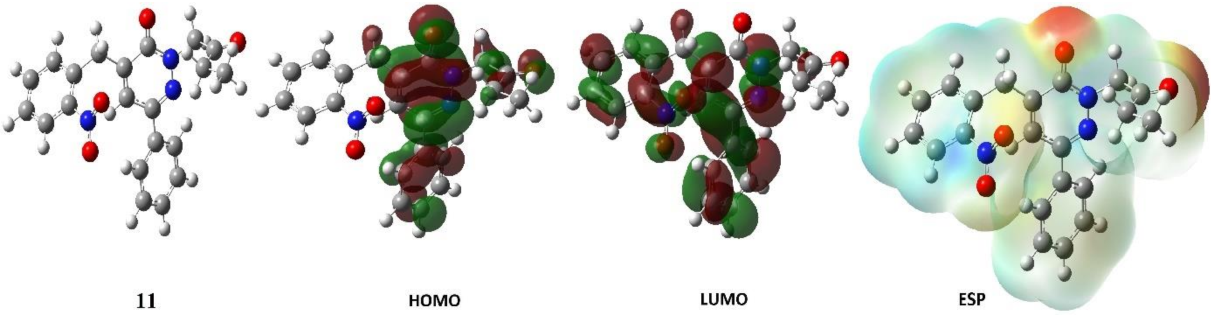

11 HOMO LUMO ESP

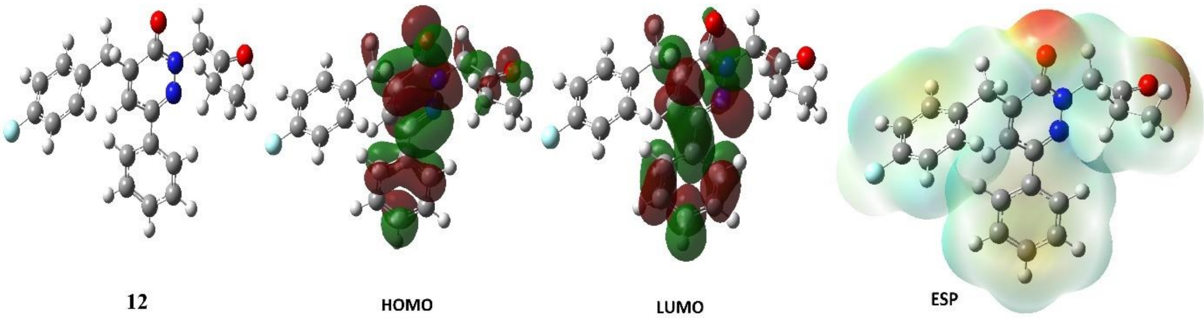

12 HOMO LUMO ESP

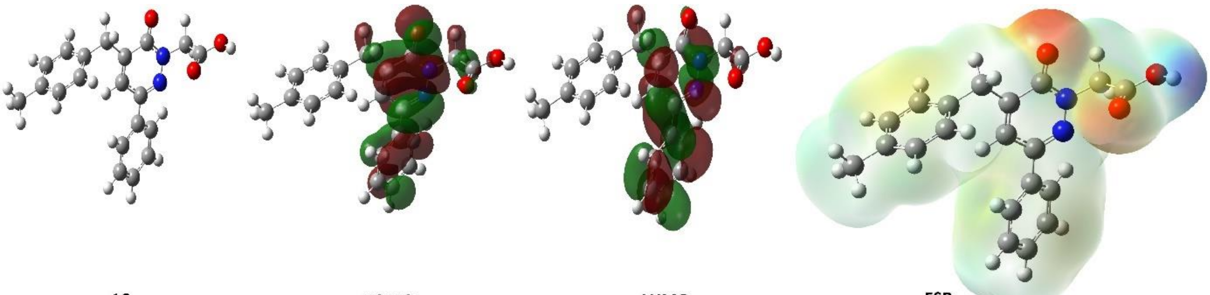

13 HOMO LUMO ESP

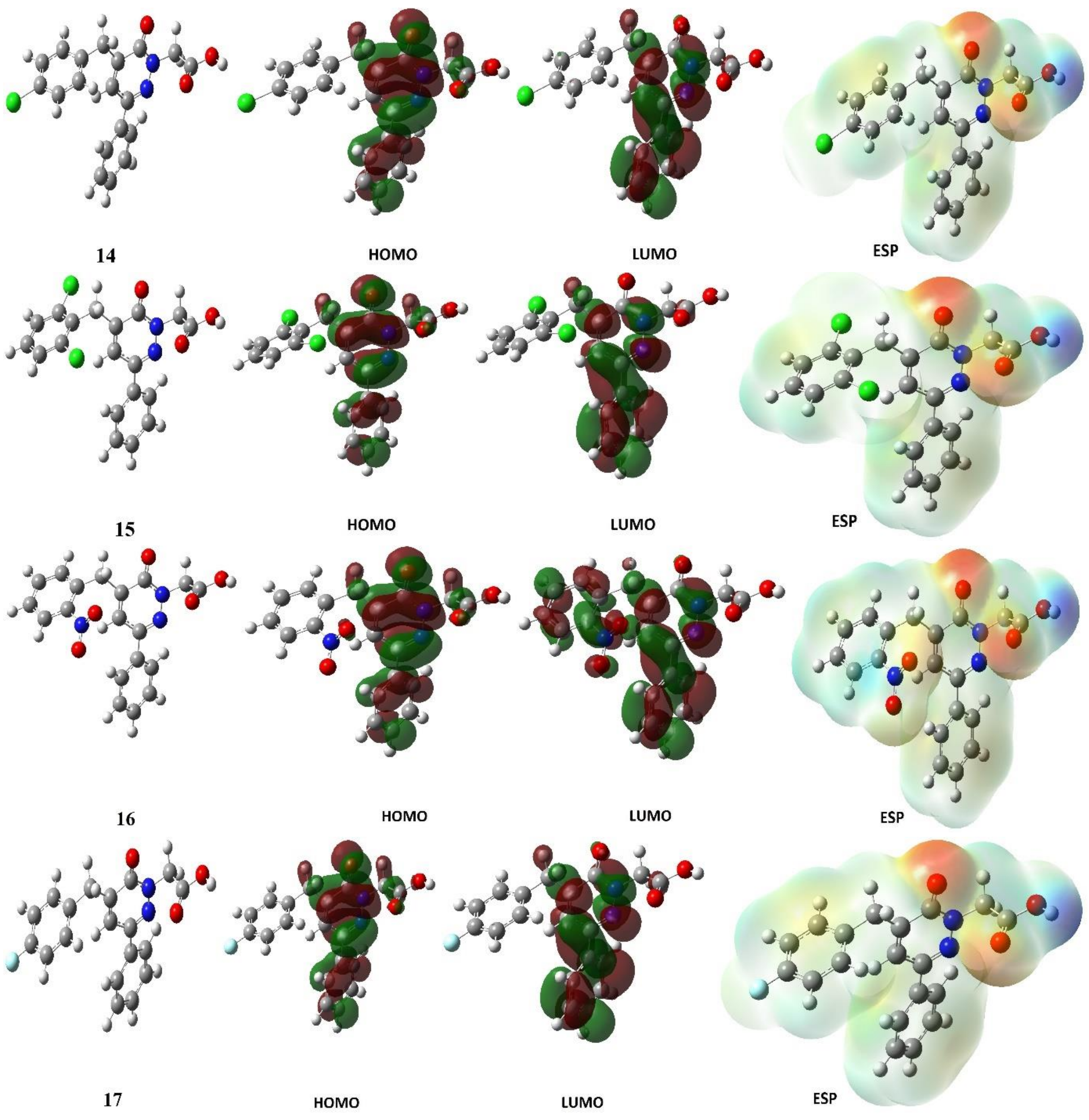

Figure S1. Shapes of optimized structure, HOMO, LUMO and ESP of all molecules.

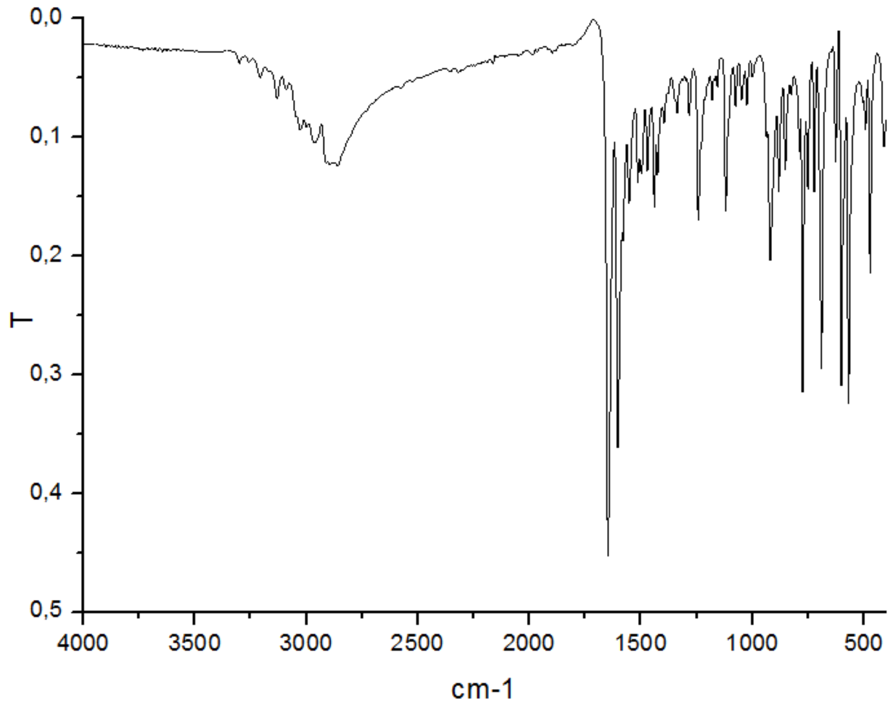

Figure S2. FT-IR spectrum of **3.**

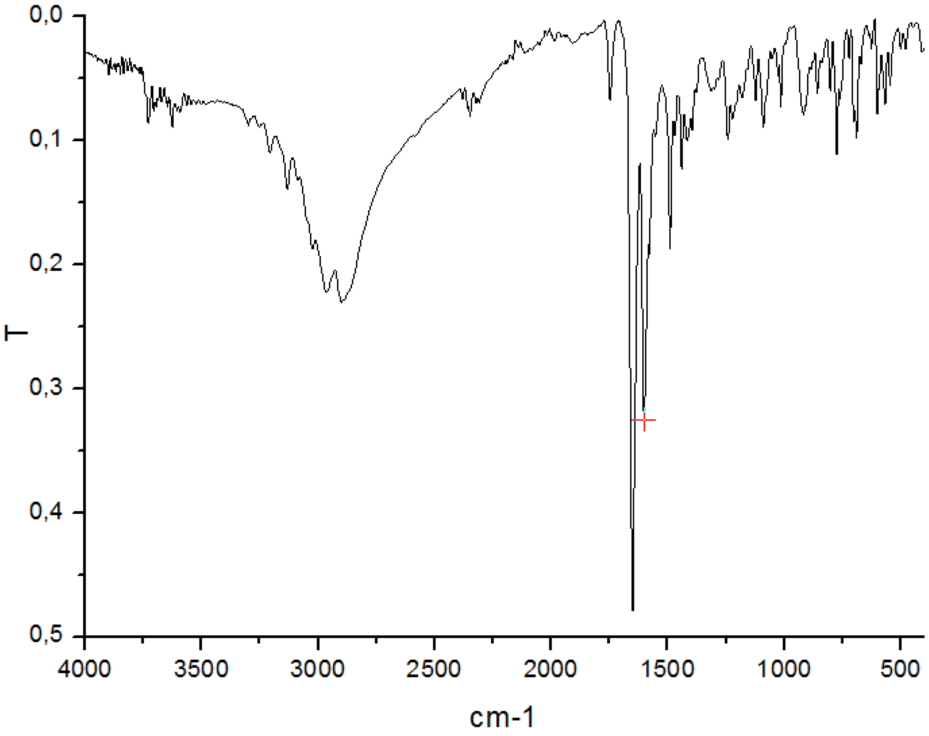

Figure S3. FT-IR spectrum of **4.**

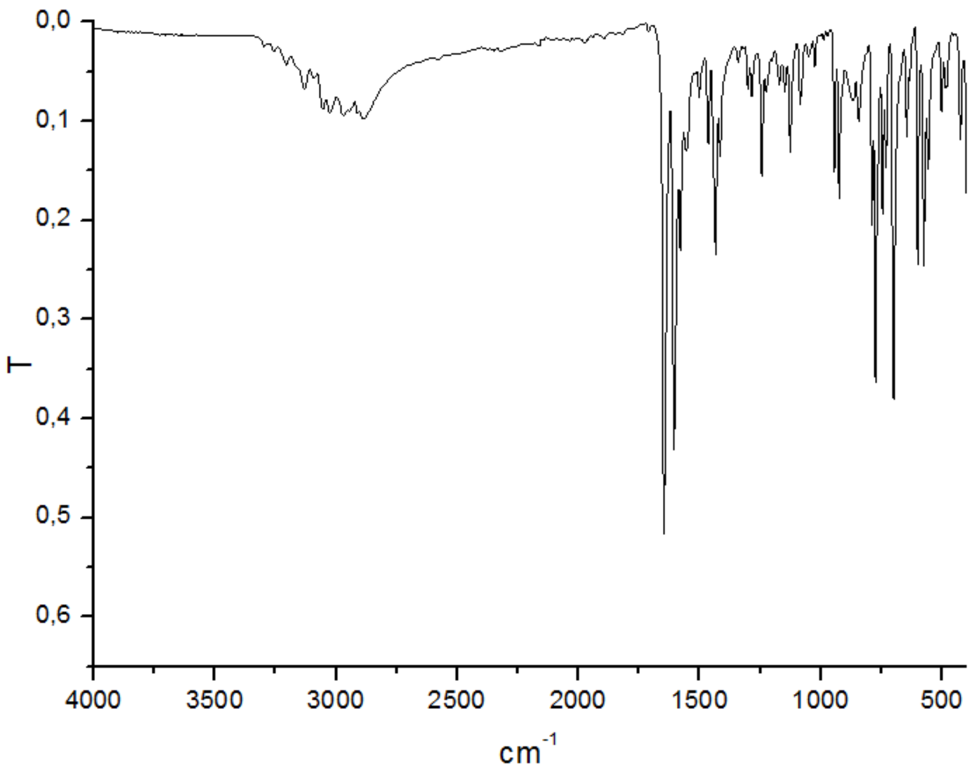

Figure S4. FT-IR spectrum of **5.**

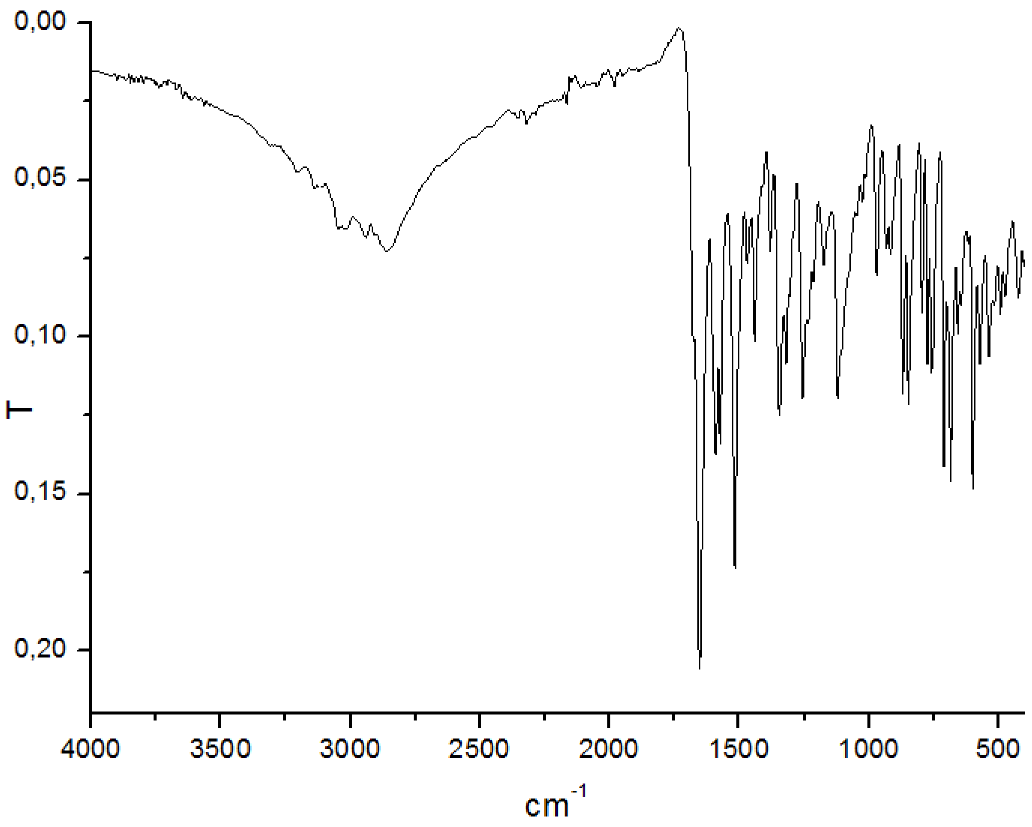

Figure S5. FT-IR spectrum of **6.**

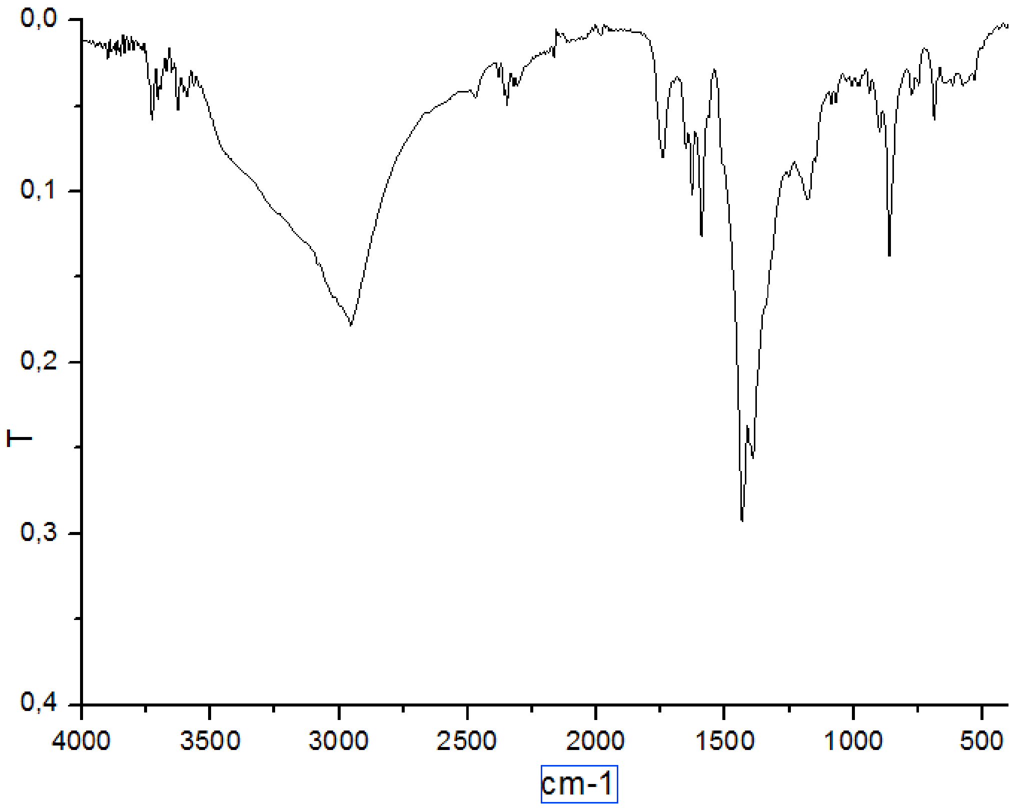

Figure S6. FT-IR spectrum of **7.**

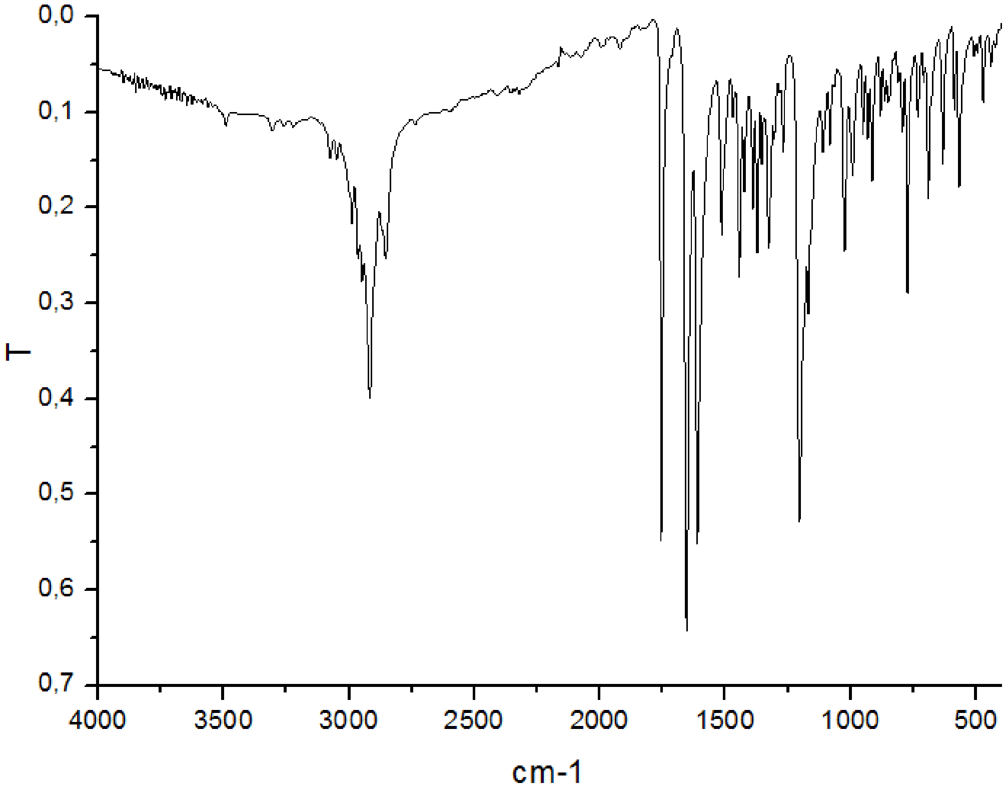

Figure S7. FT-IR spectrum of **8.**

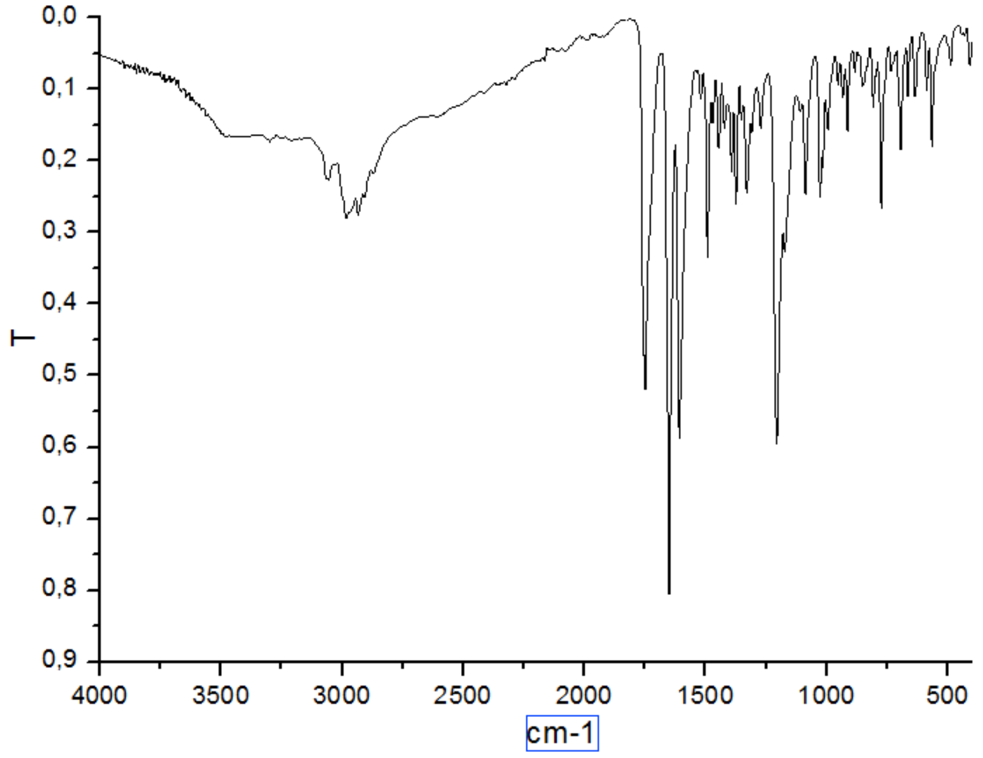

Figure S8. FT-IR spectrum of **9.**

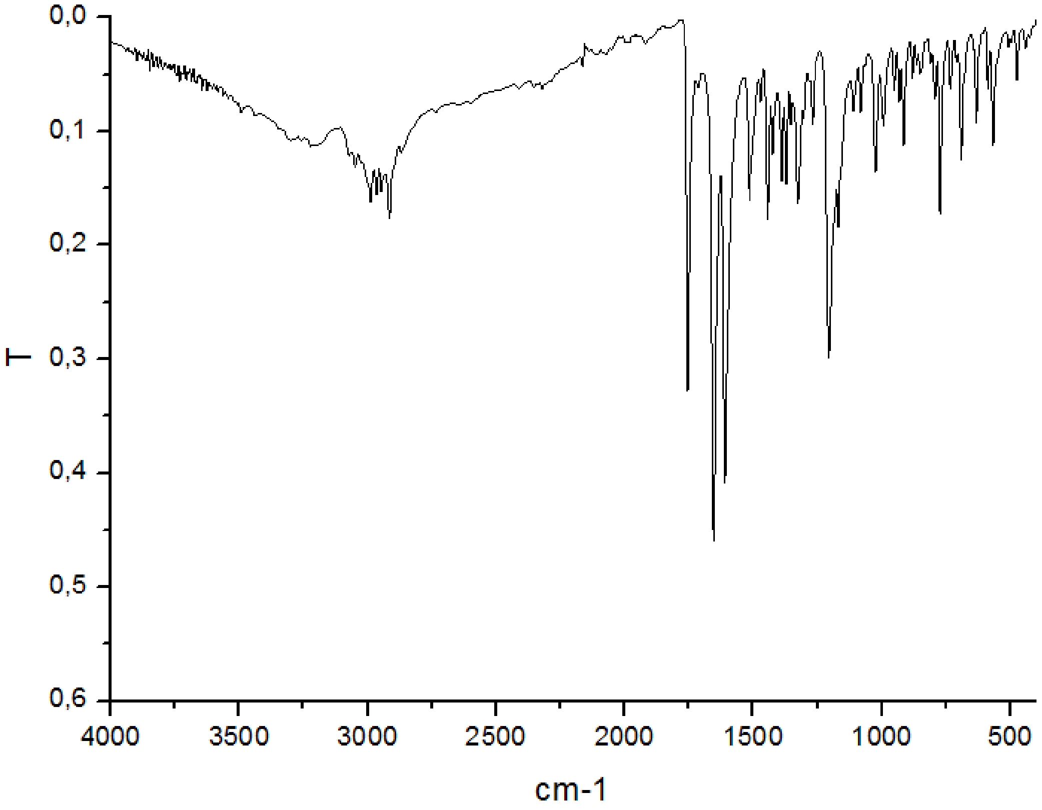

Figure S9. FT-IR spectrum of **10.**

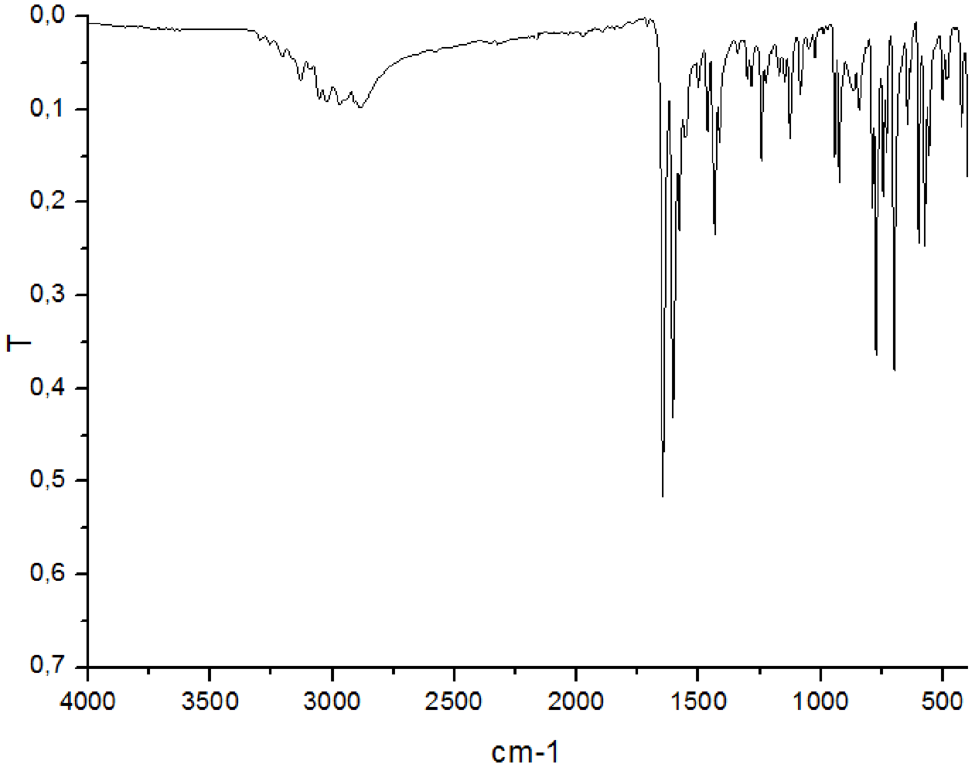

Figure S10. FT-IR spectrum of **11.**

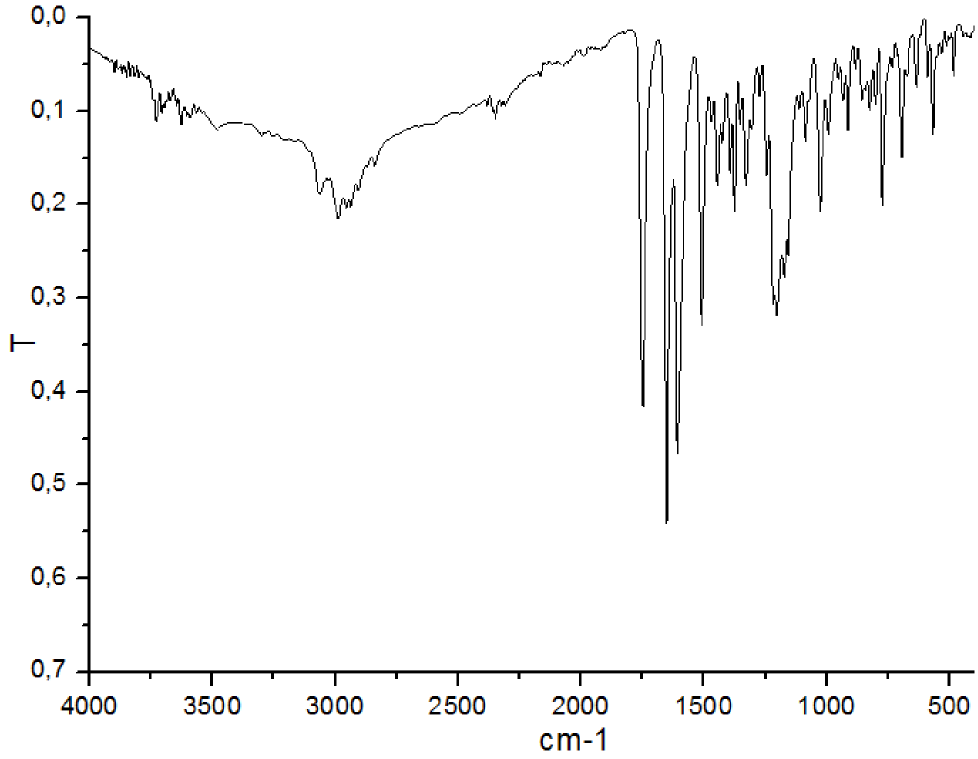

Figure S11. FT-IR spectrum of **12.**

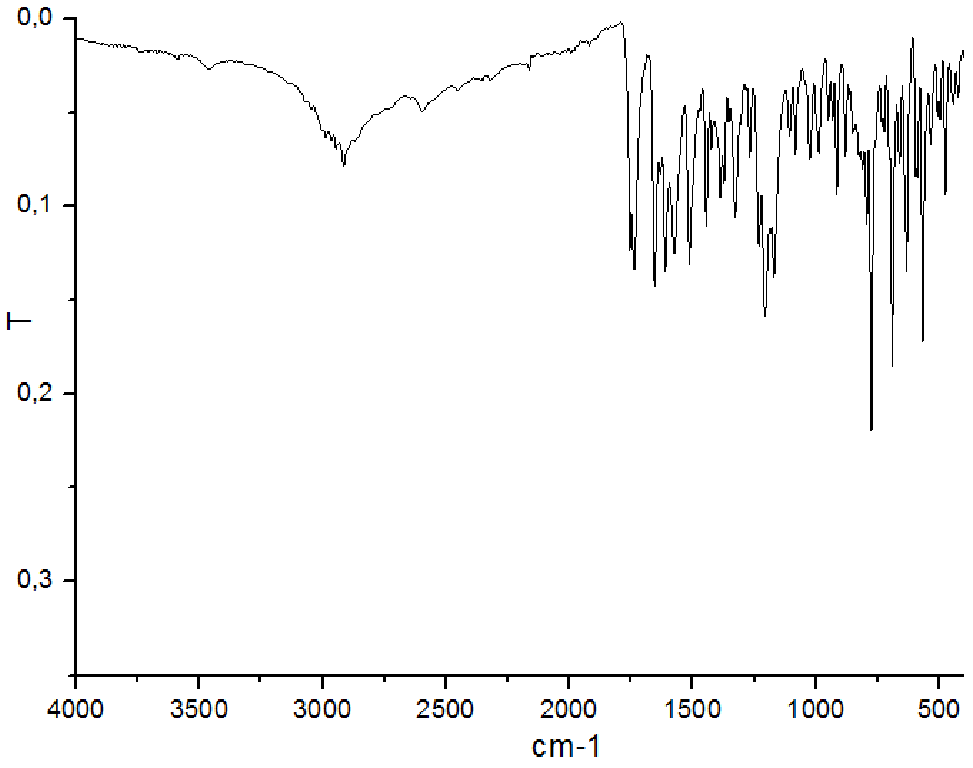

Figure S112 FT-IR spectrum of **13.**

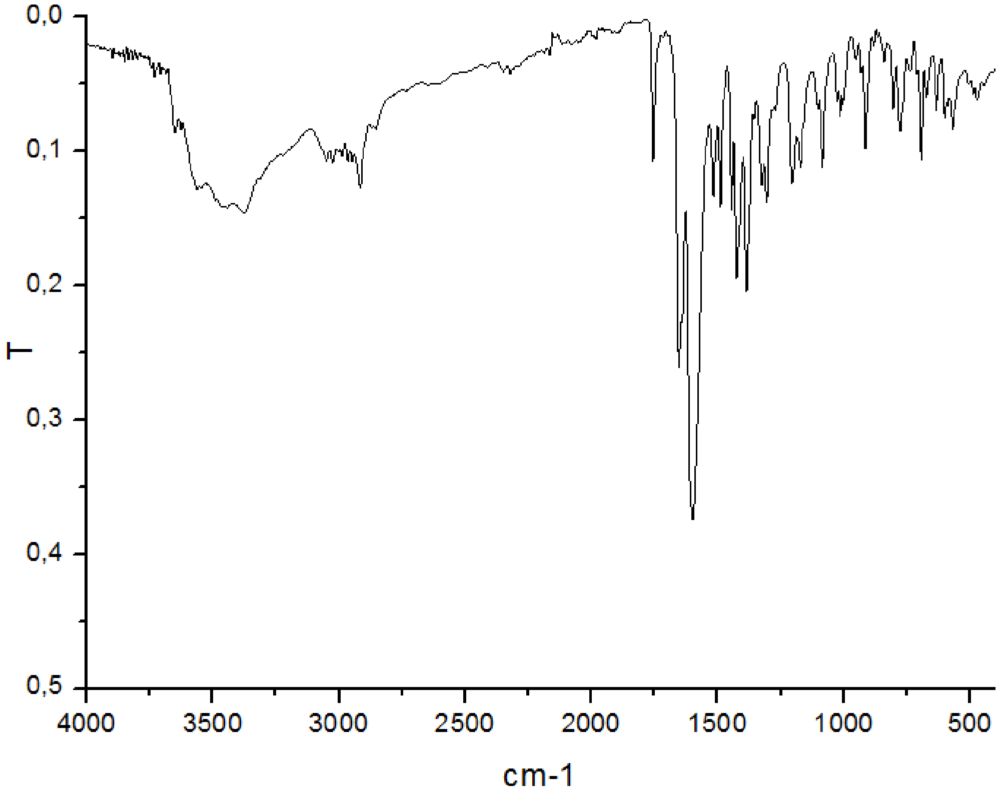

Figure S13. FT-IR spectrum of **14.**

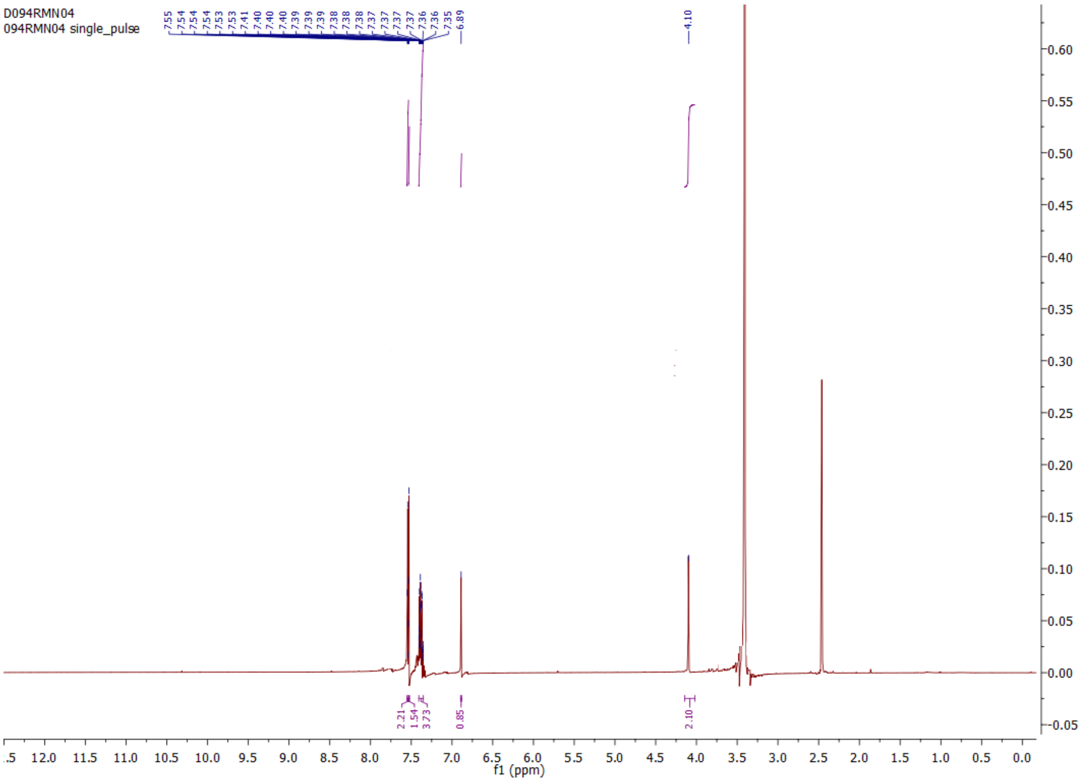

Figure S14. ^{1}H NMR spectrum of **5**.

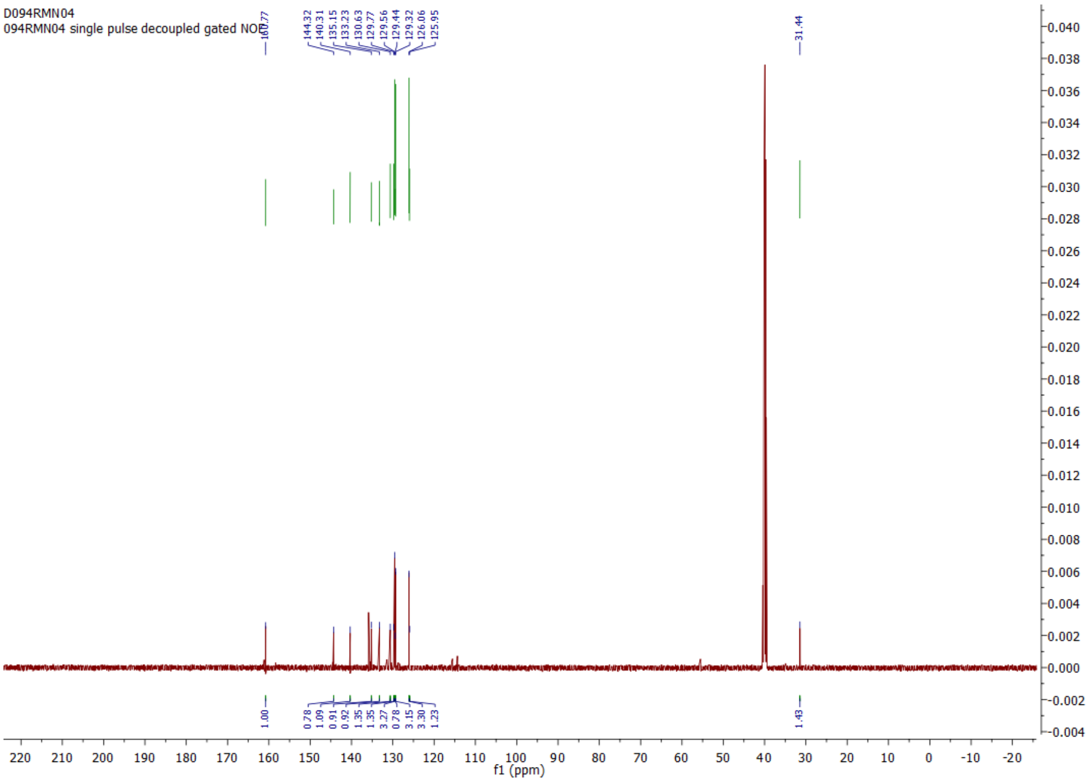

Figure S15. ^{13}C NMR spectrum of **5**.

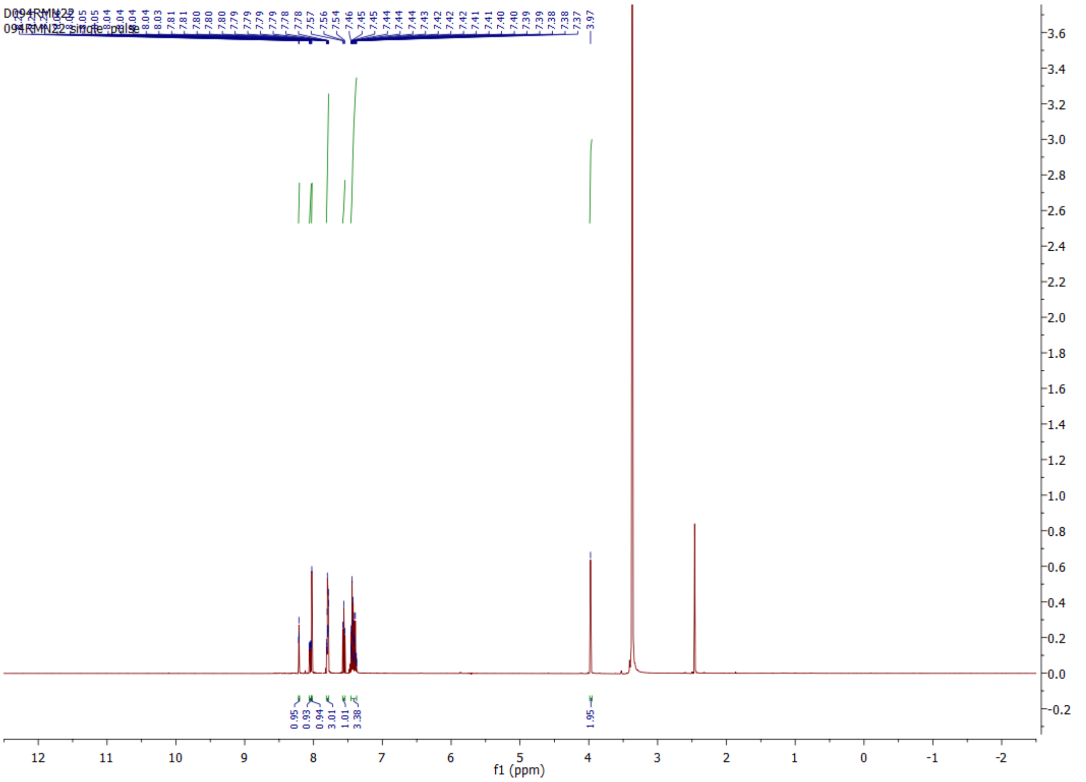

Figure S16. ^{1}H NMR spectrum of **6**.

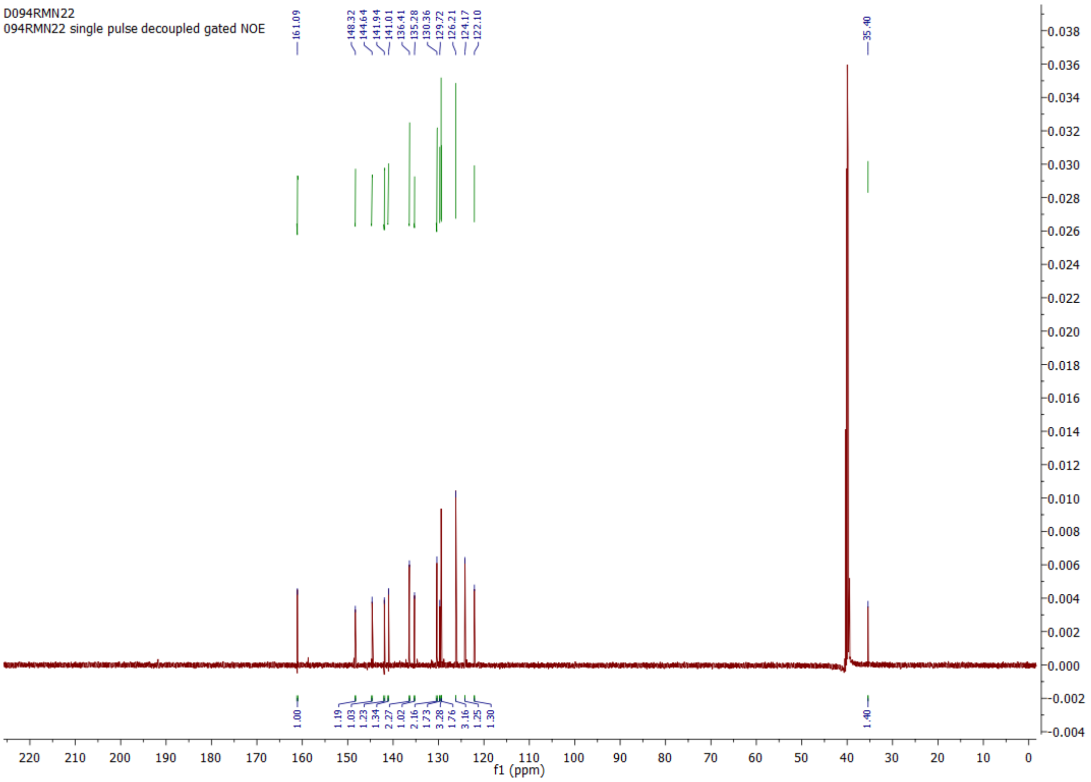

Figure S17. ^{13}C NMR spectrum of **6**.

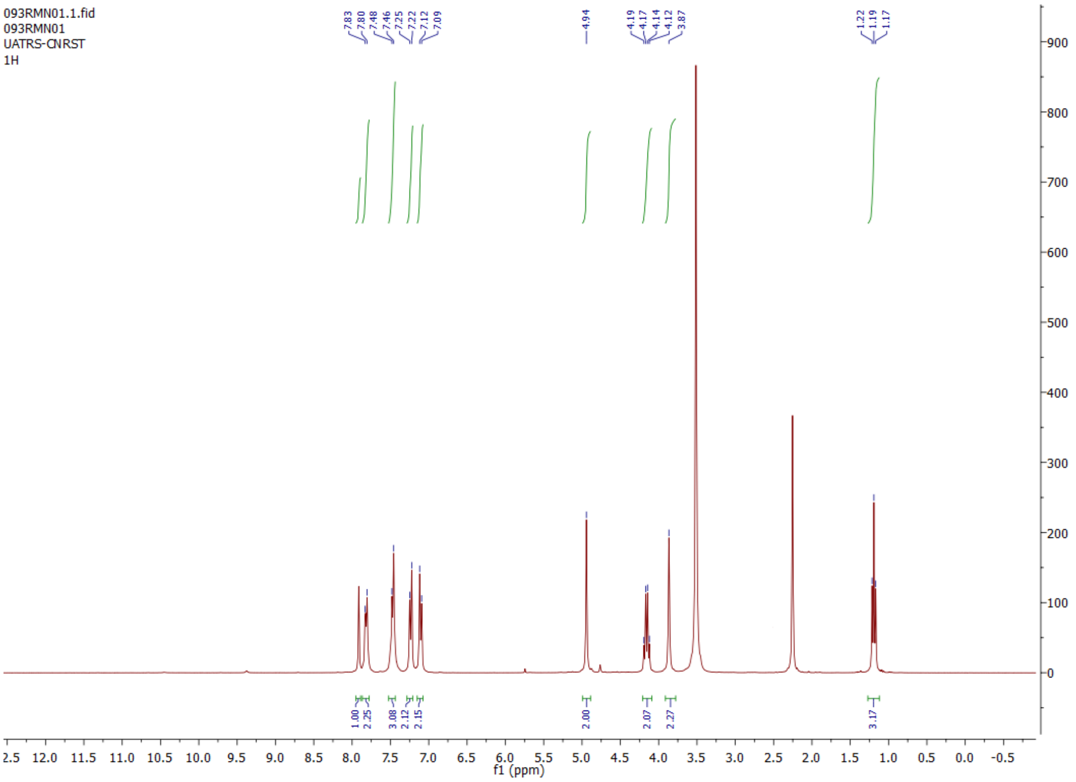

Figure S18. ^{1}H NMR spectrum of **8**.

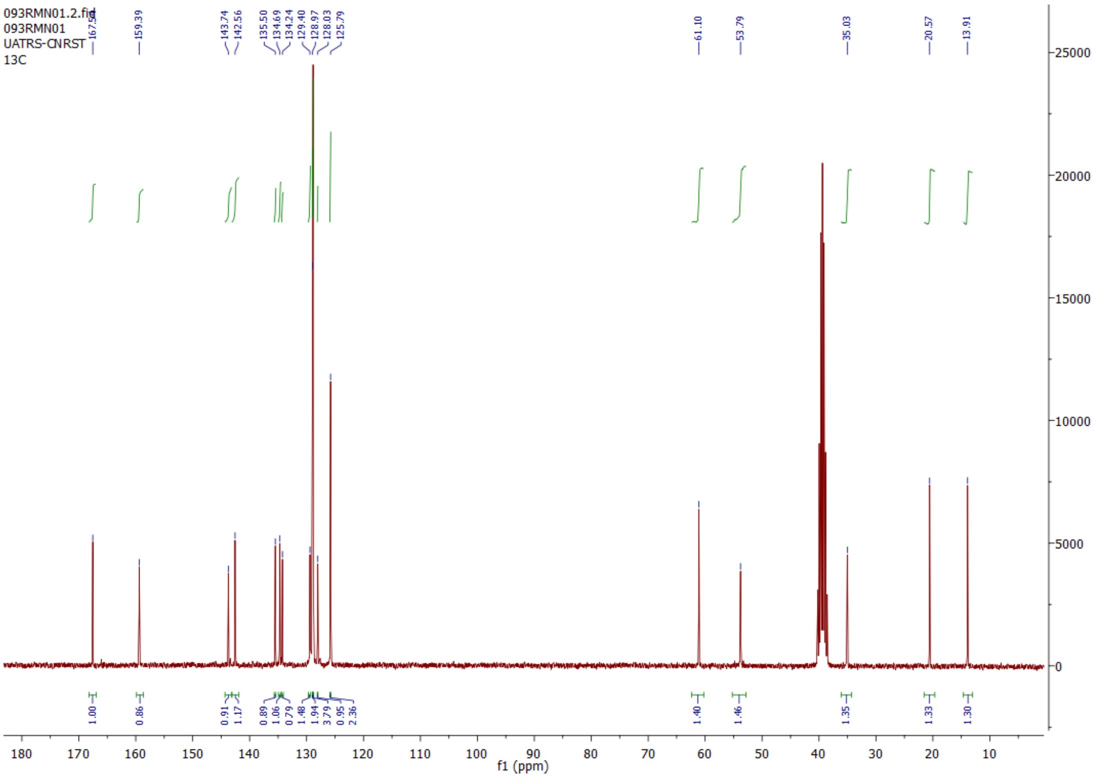

Figure S19. ^{13}C NMR spectrum of **8**.

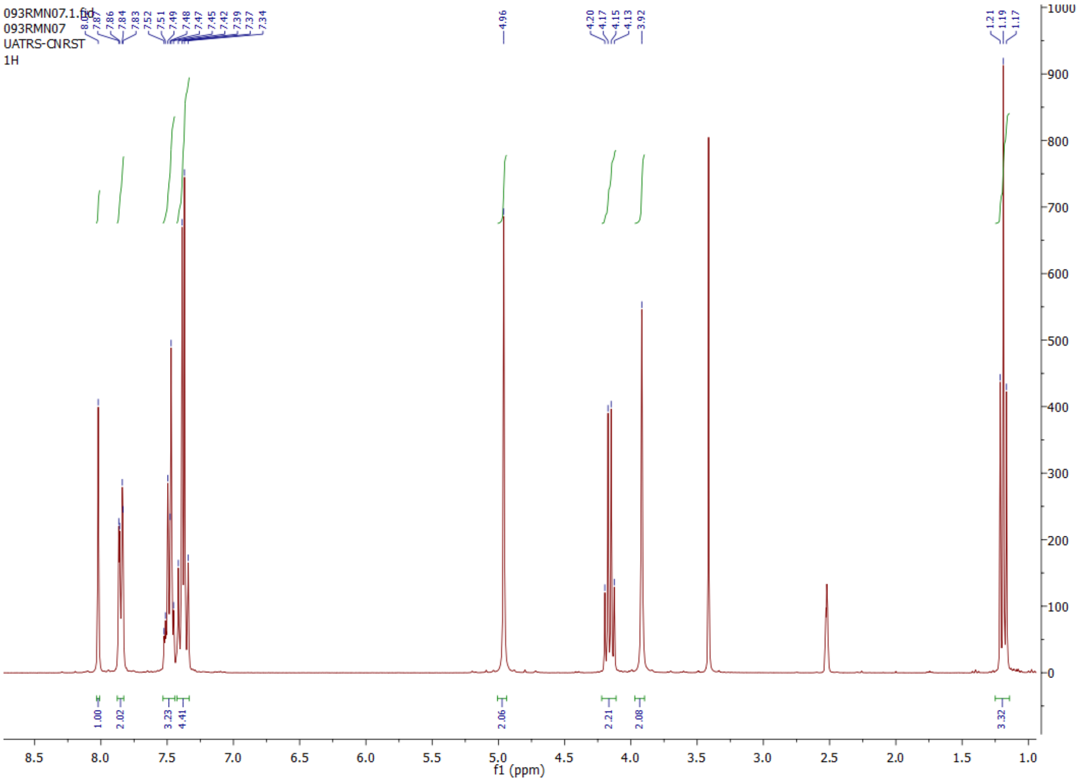

Figure S20. ^{1}H NMR spectrum of **9**.

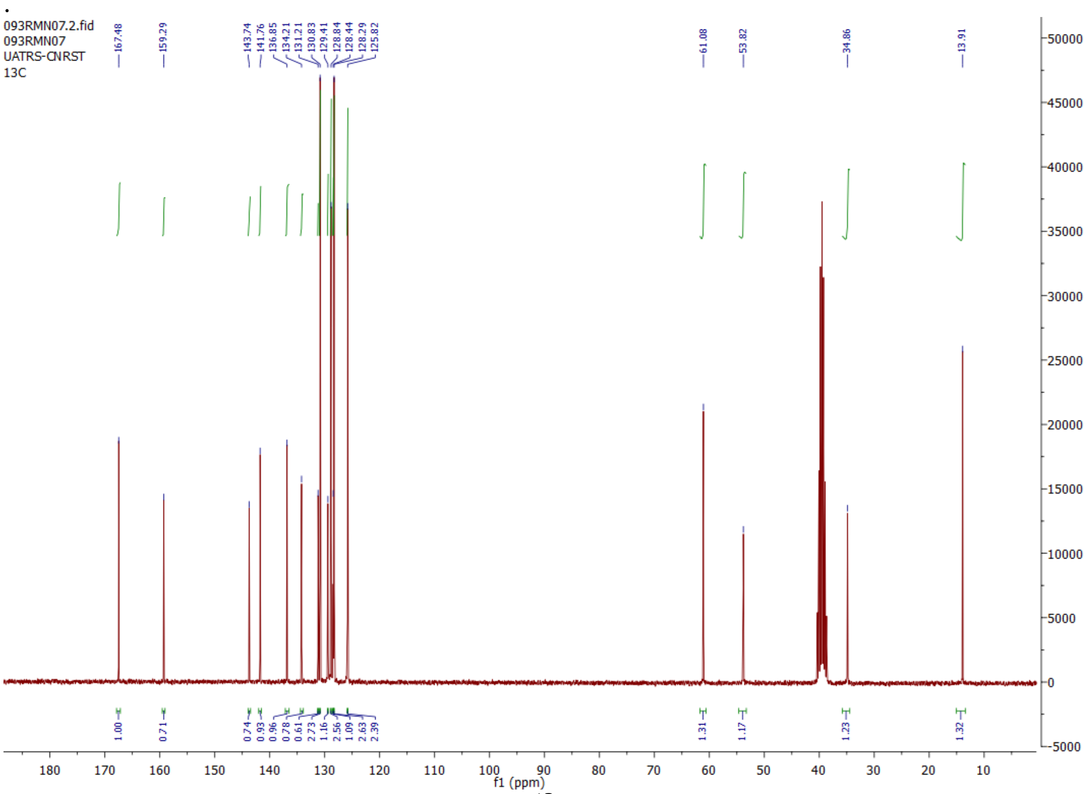

Figure S21. ^{13}C NMR spectrum of **9**.

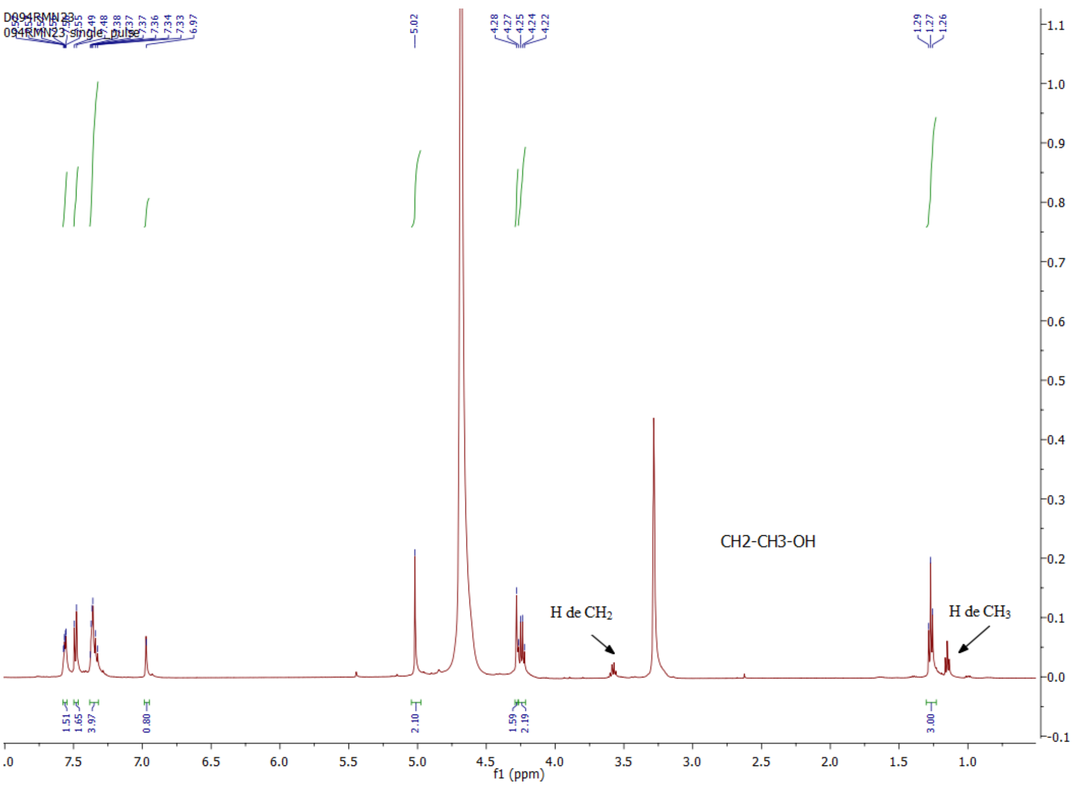

Figure S22. ^{1}H NMR spectrum of **10**.

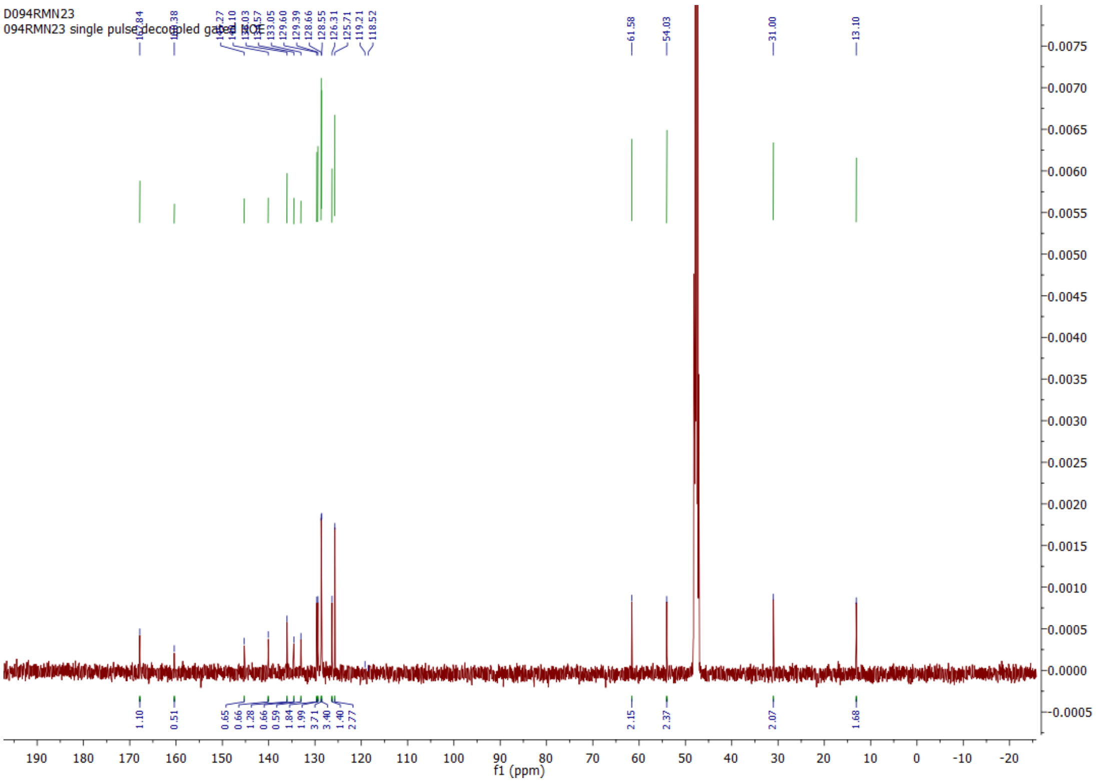

Figure S23. ^{13}C NMR spectrum of **10**.

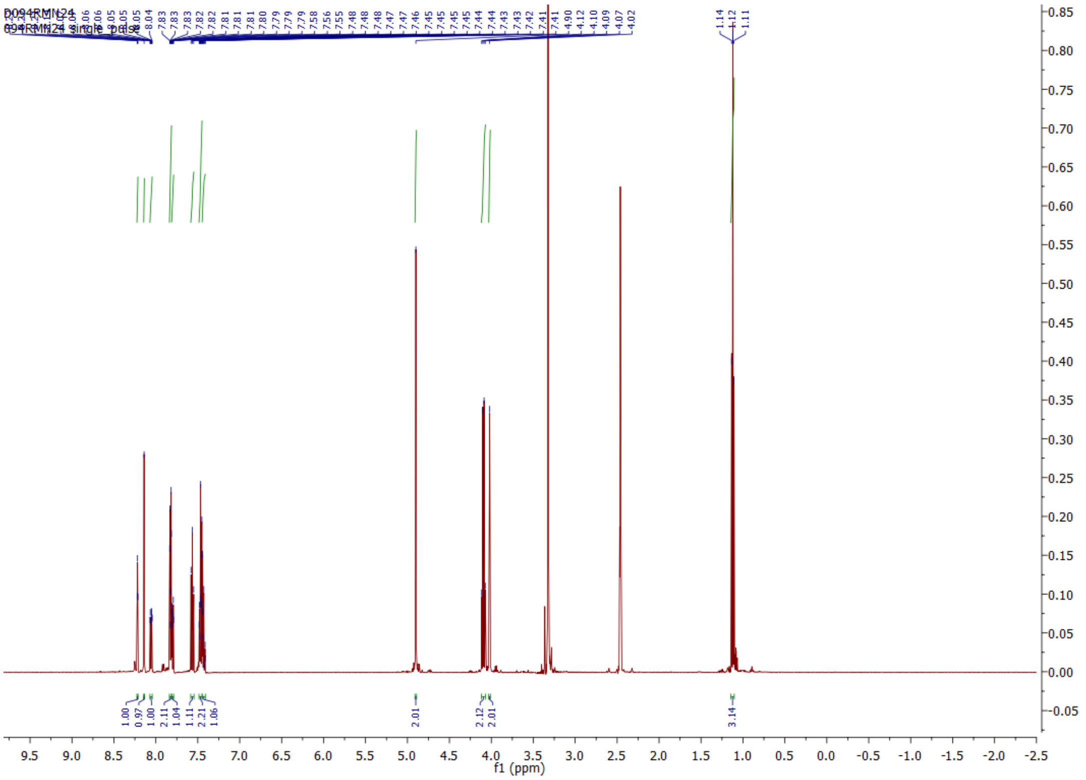

Figure S24. ^{1}H NMR spectrum of **11**.

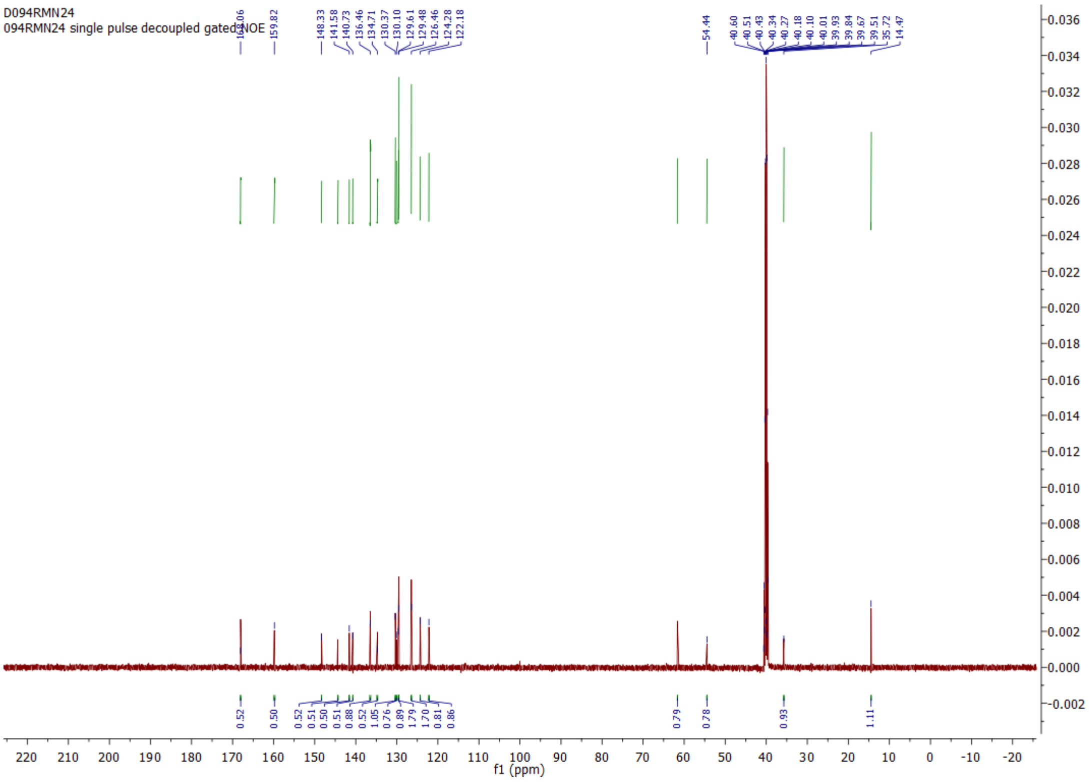

Figure S25. ^{13}C NMR spectrum of **11**.

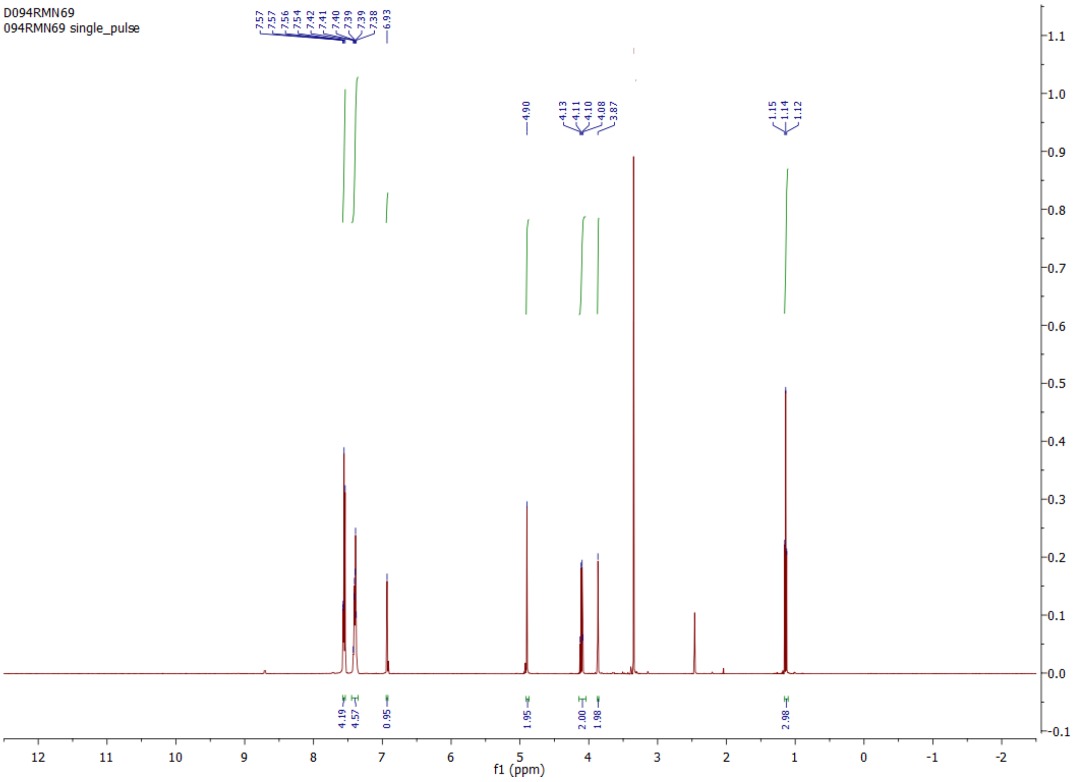

Figure S26. ^{1}H NMR spectrum of **12**.

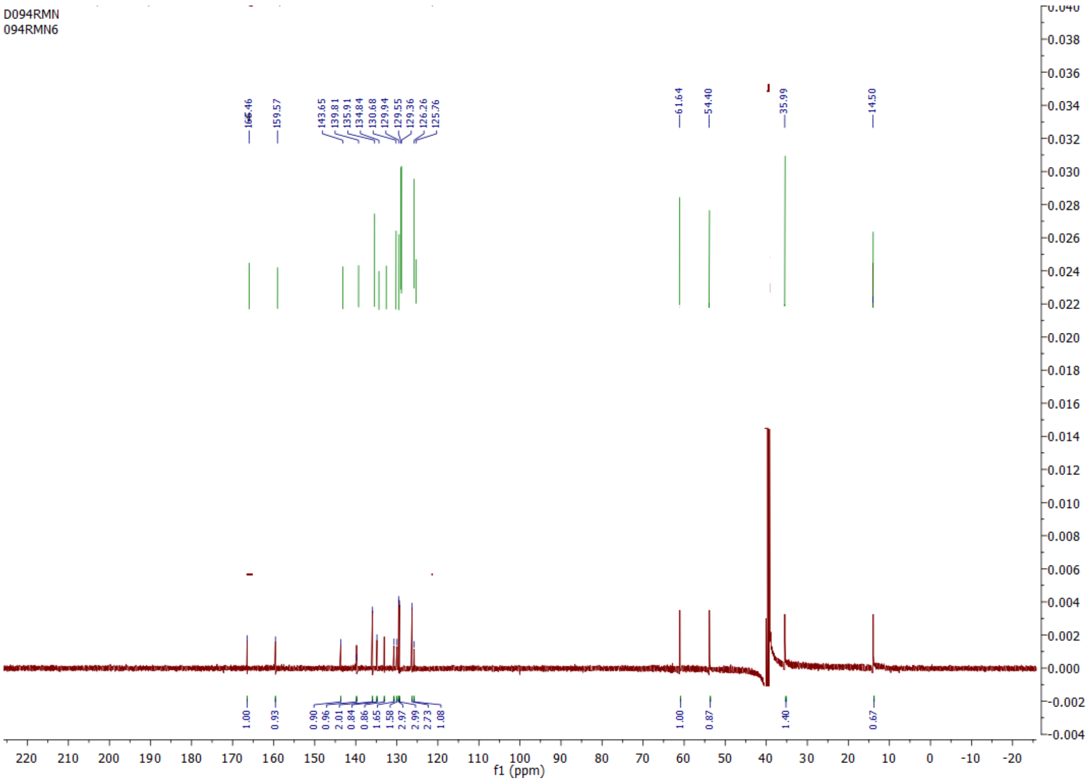

Figure S27. ^{13}C NMR spectrum of **12**.

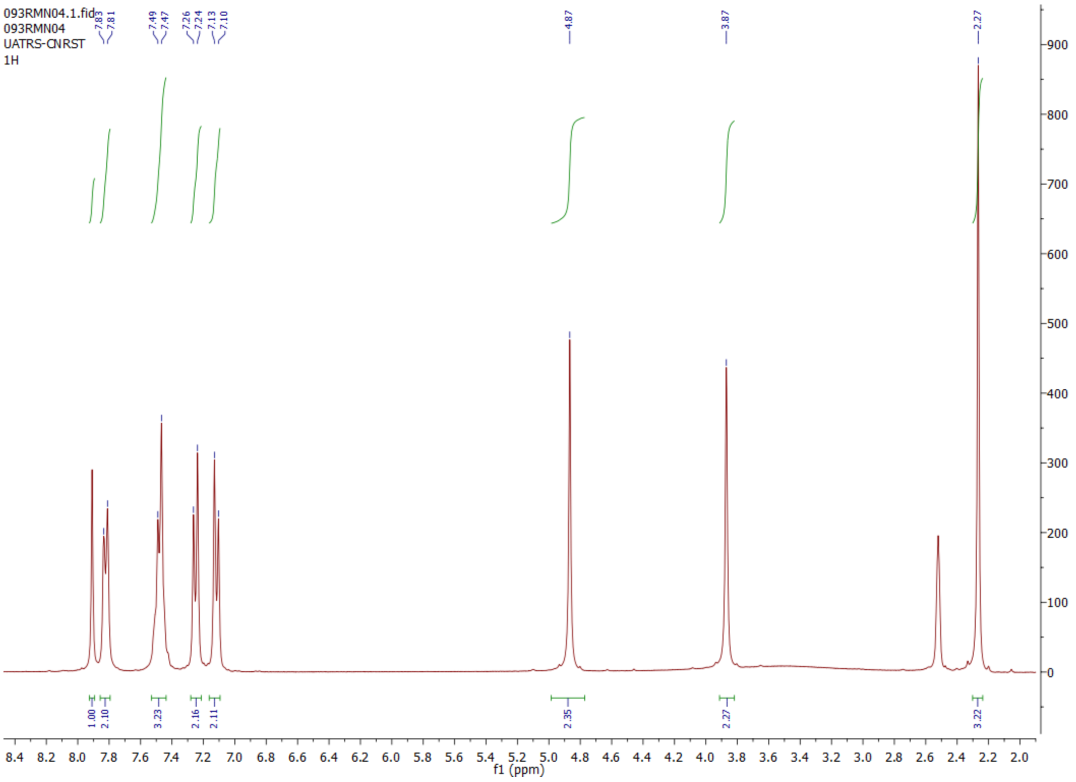

Figure S28. ^{1}H NMR spectrum of **13**.

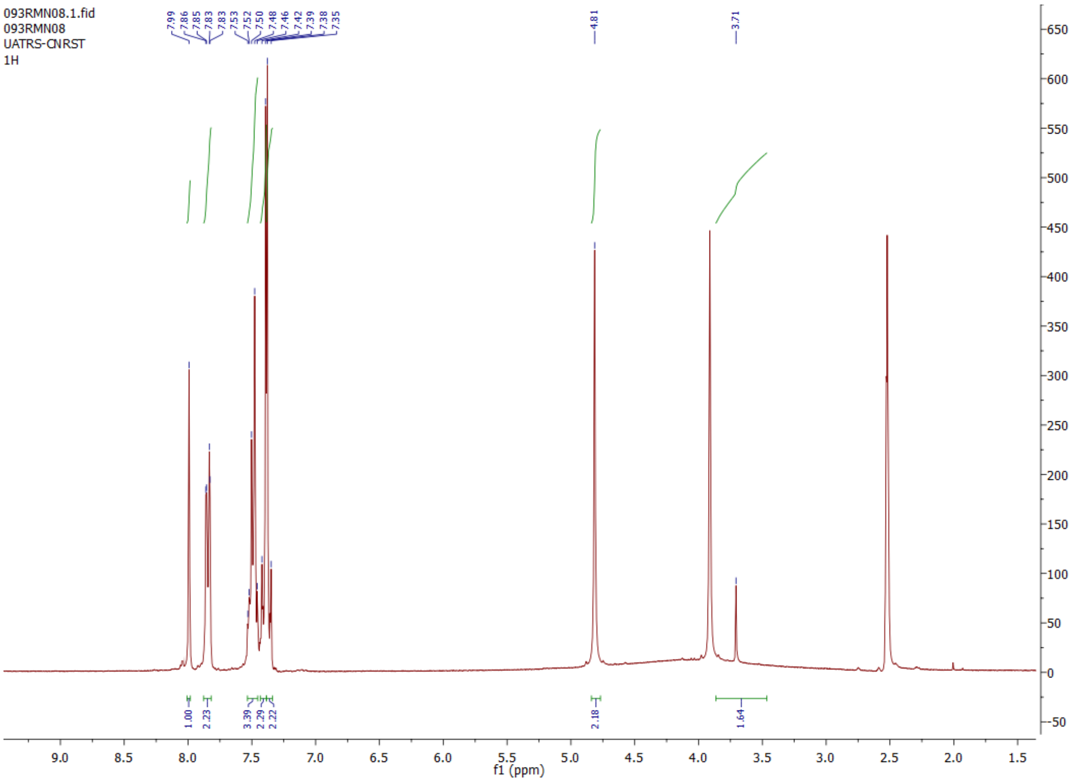

Figure S29. ^{1}H NMR spectrum of **14**.

.

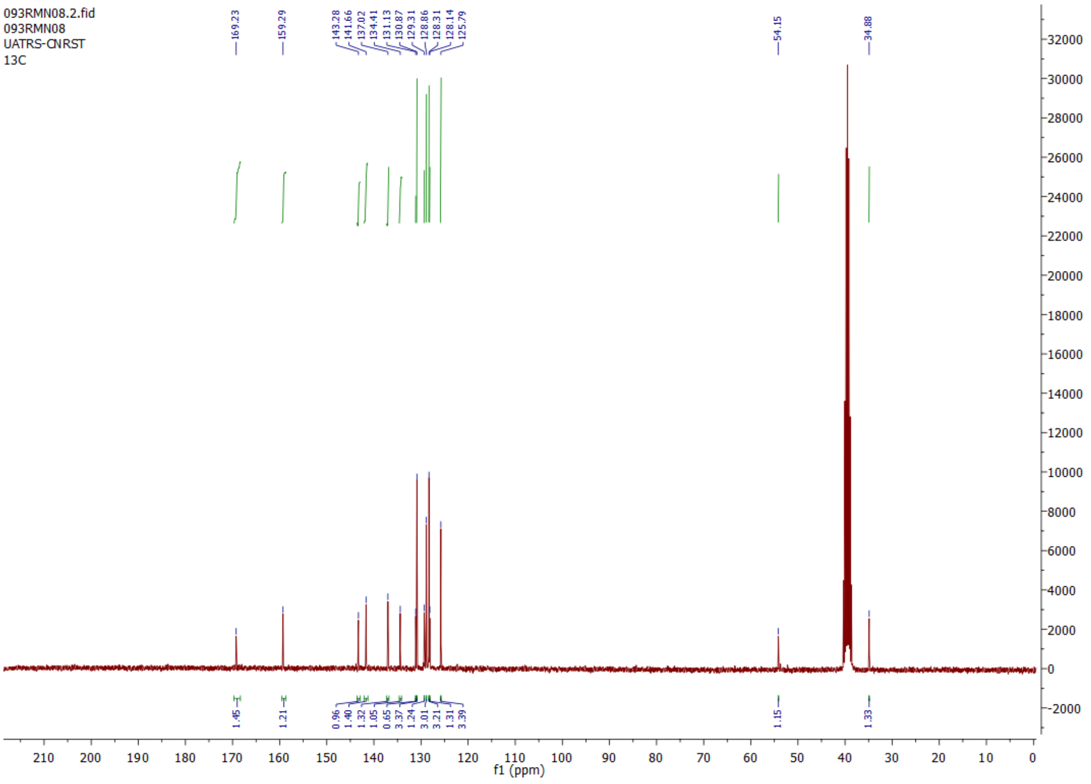

Figure S30. ^{13}C NMR spectrum of **14**.

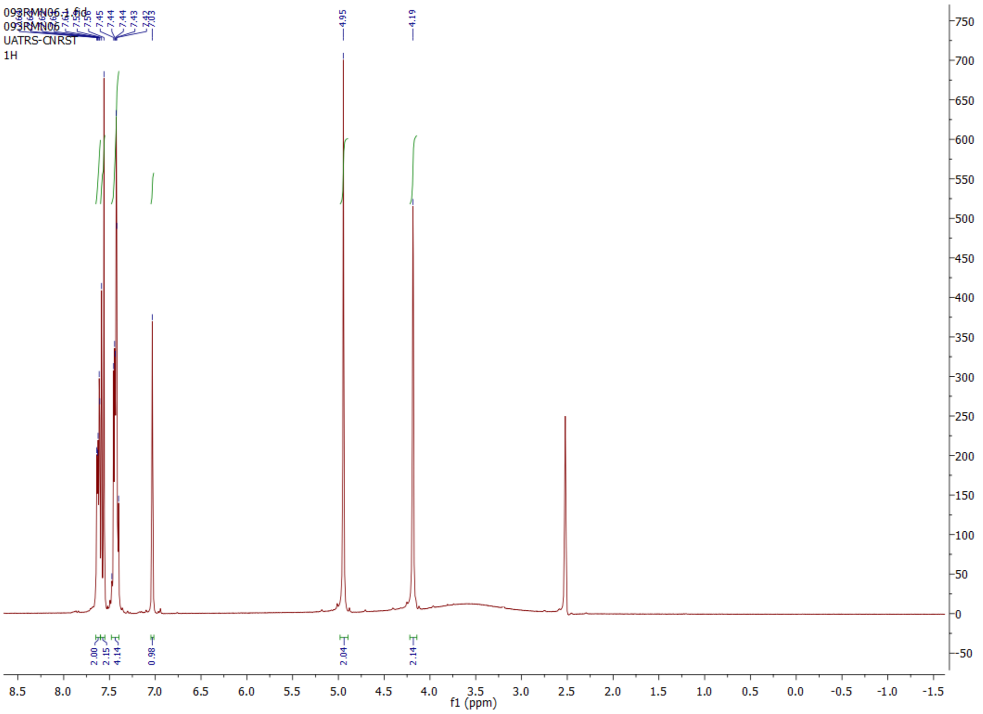

Figure S31. ^{1}H NMR spectrum of **15**.

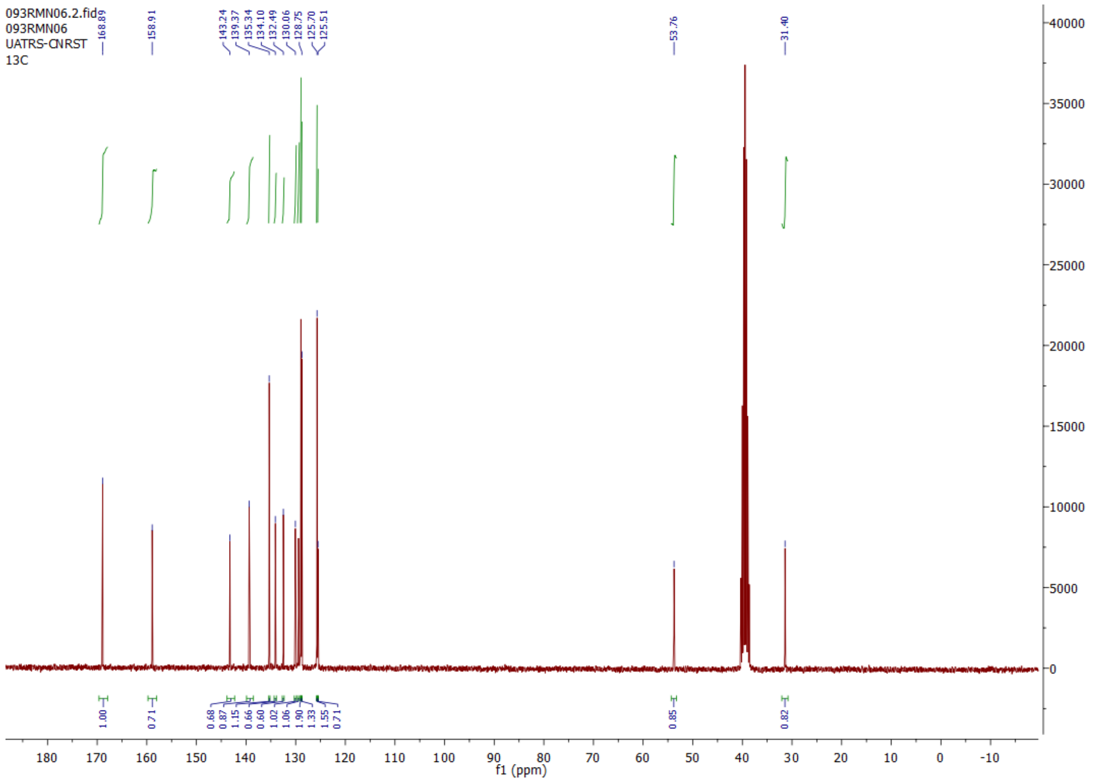

Figure S32. ^{13}C NMR spectrum of **15**.

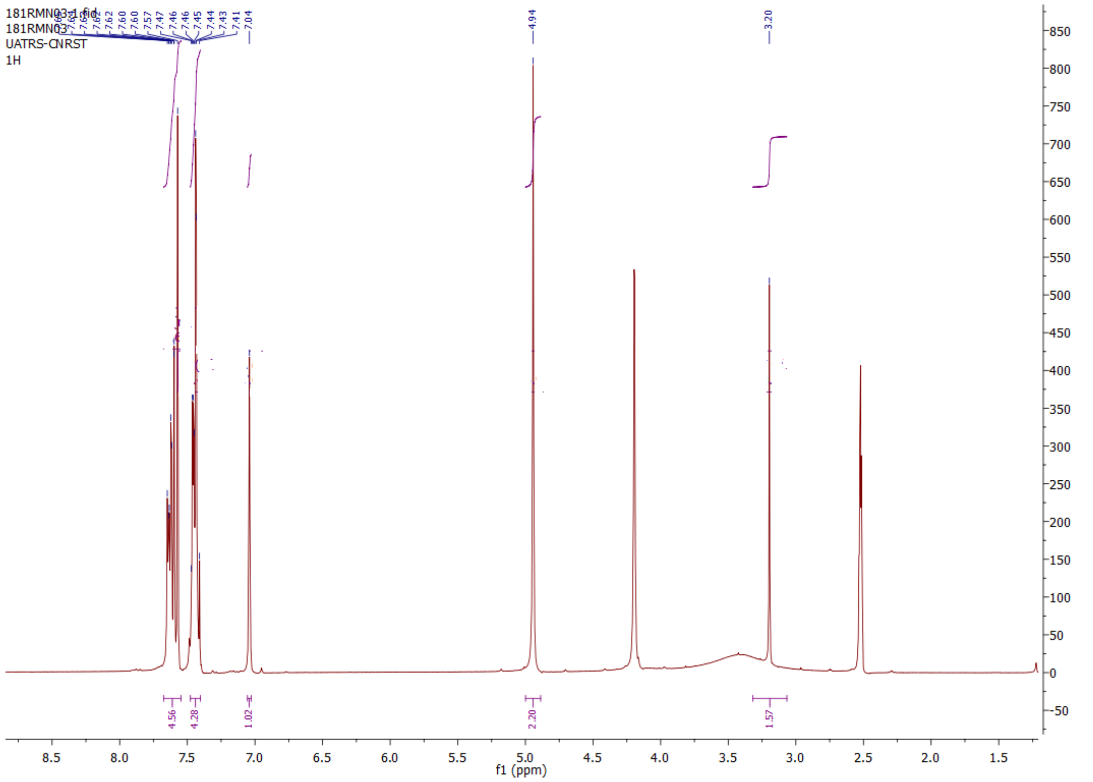

Figure S33. ^{1}H NMR spectrum of **17**.

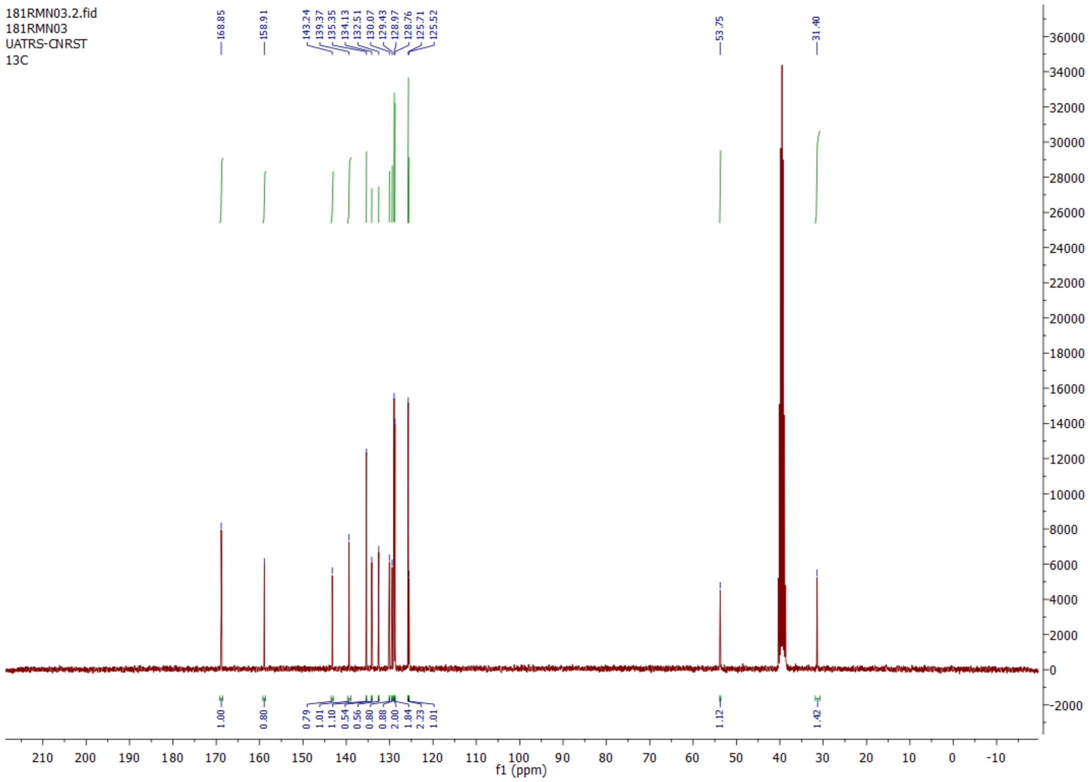

Figure S34. ^{13}C NMR spectrum of **17**.

Copies of HRMS spectra

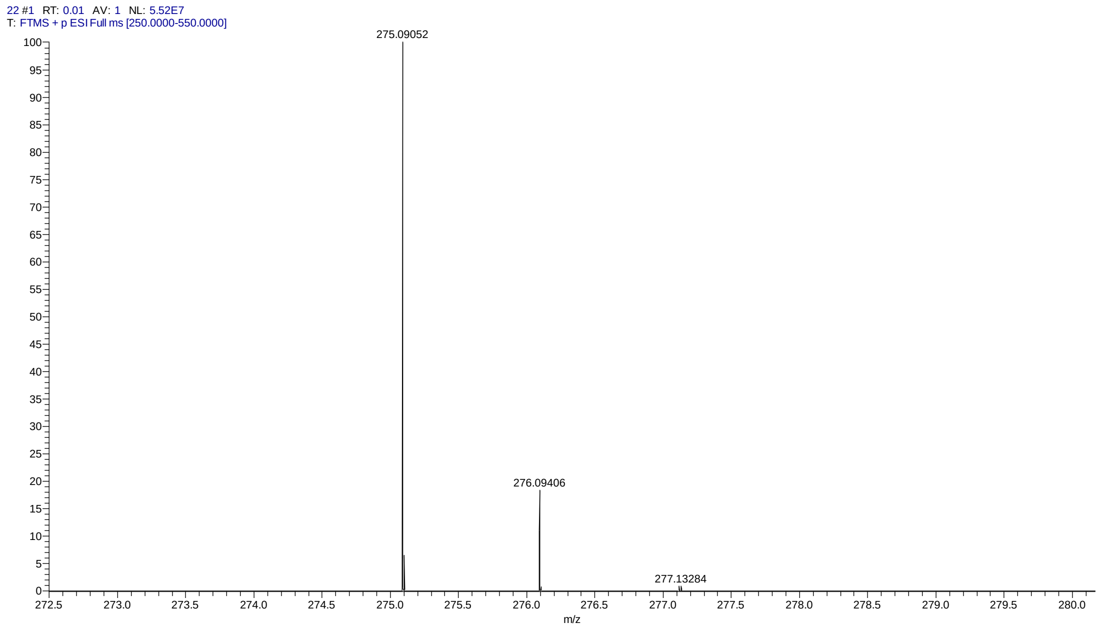

Figure S35. ESI-MS spectrum of **3**.

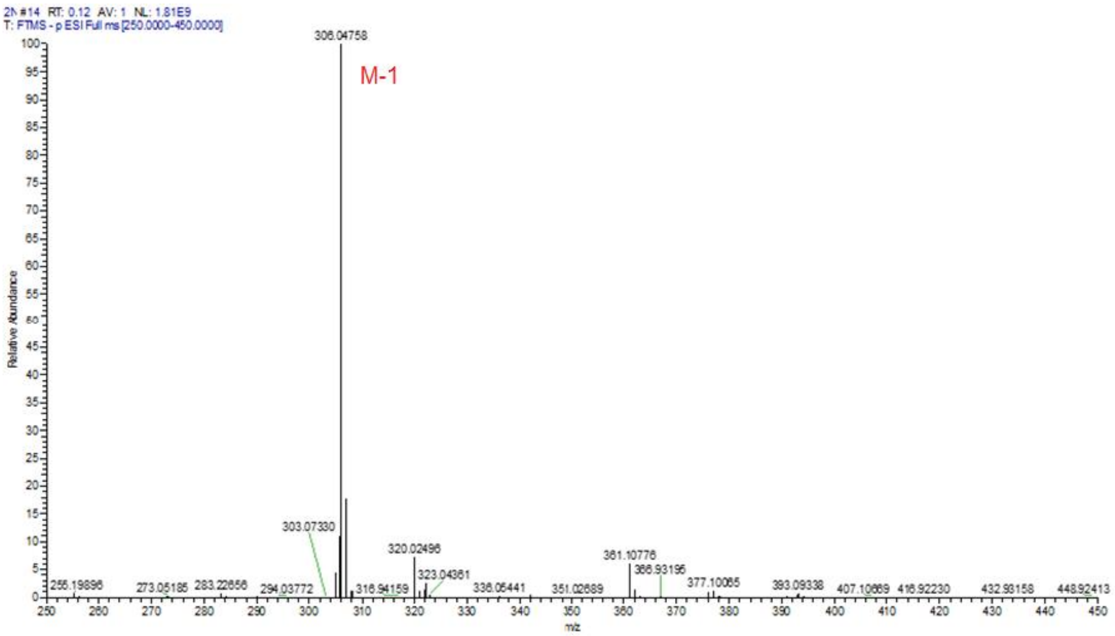

Figure S36. ESI-HRMS spectrum of **6**.

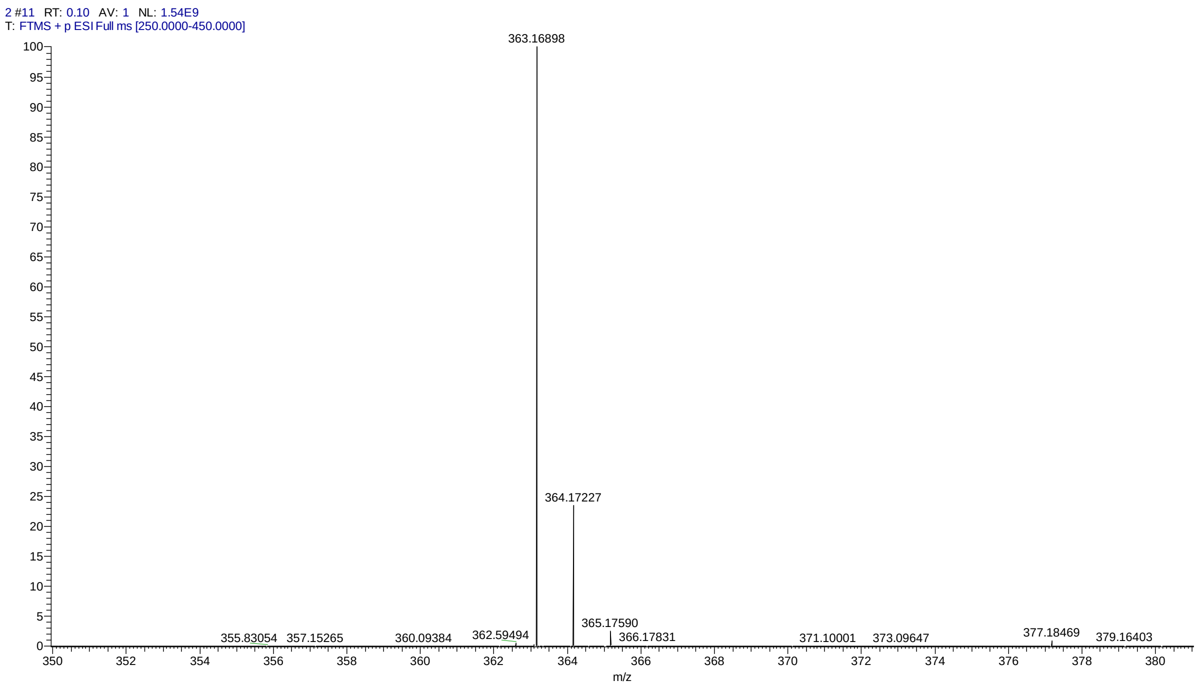

Figure S37. ESI-HRMS spectrum of **8**.

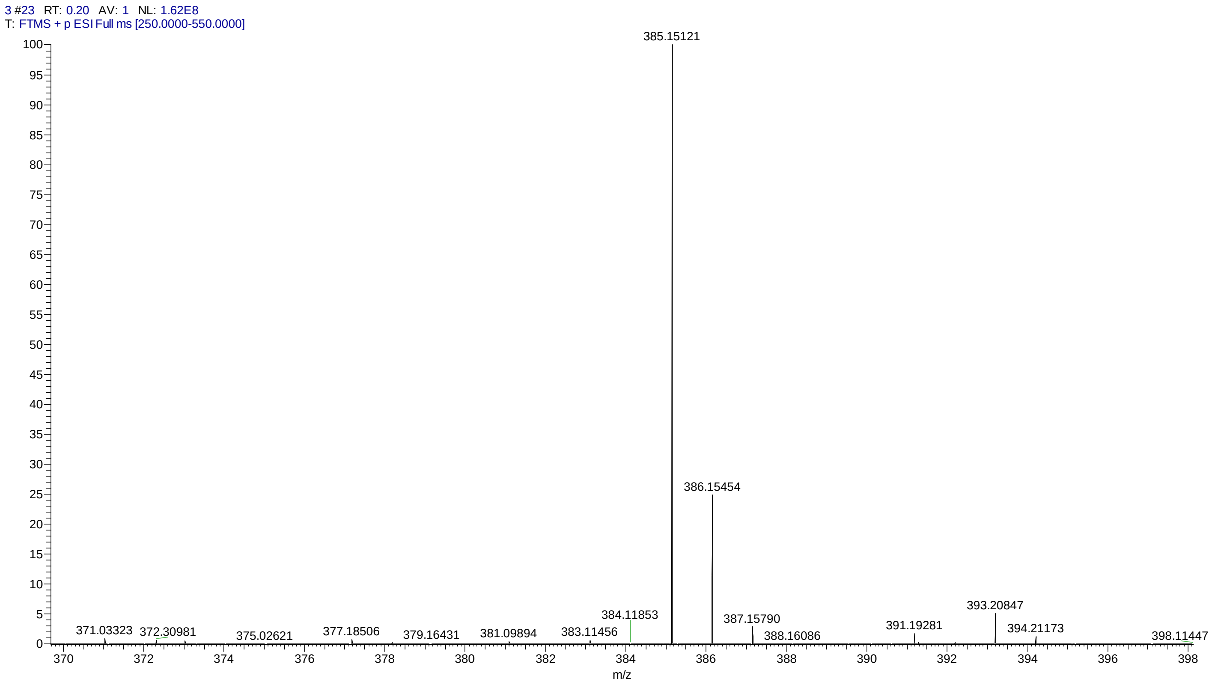

Figure S38. ESI-HRMS spectrum of **9**.

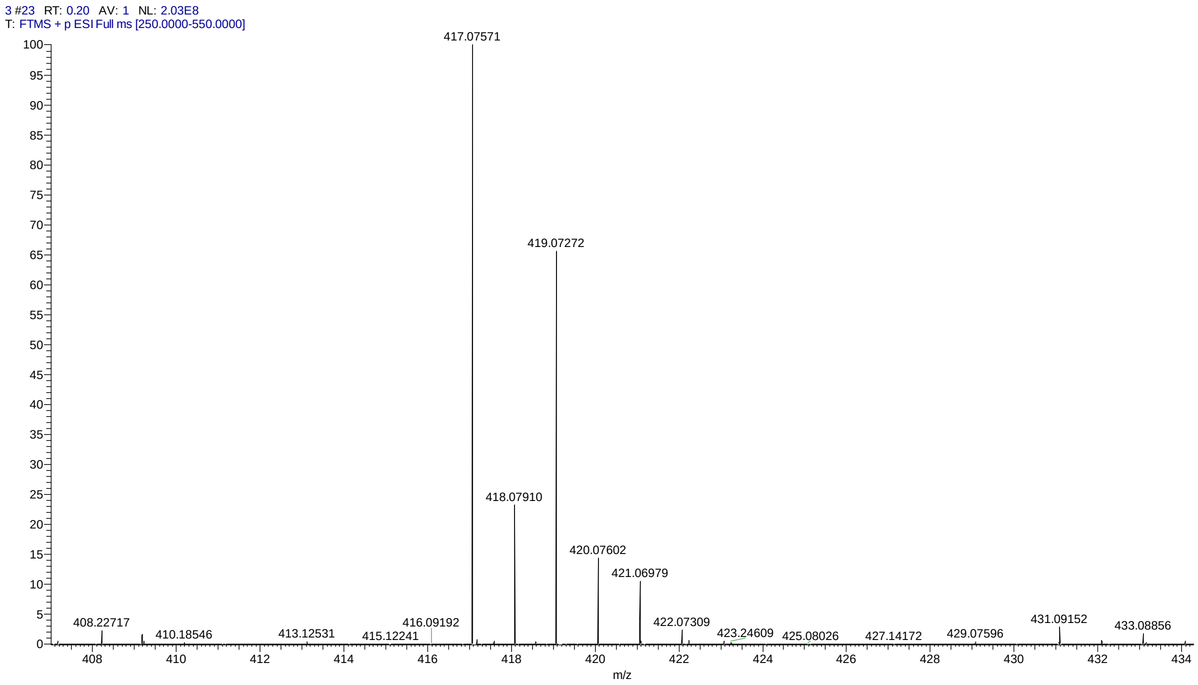

Figure S39. ESI-HRMS spectrum of **10**.

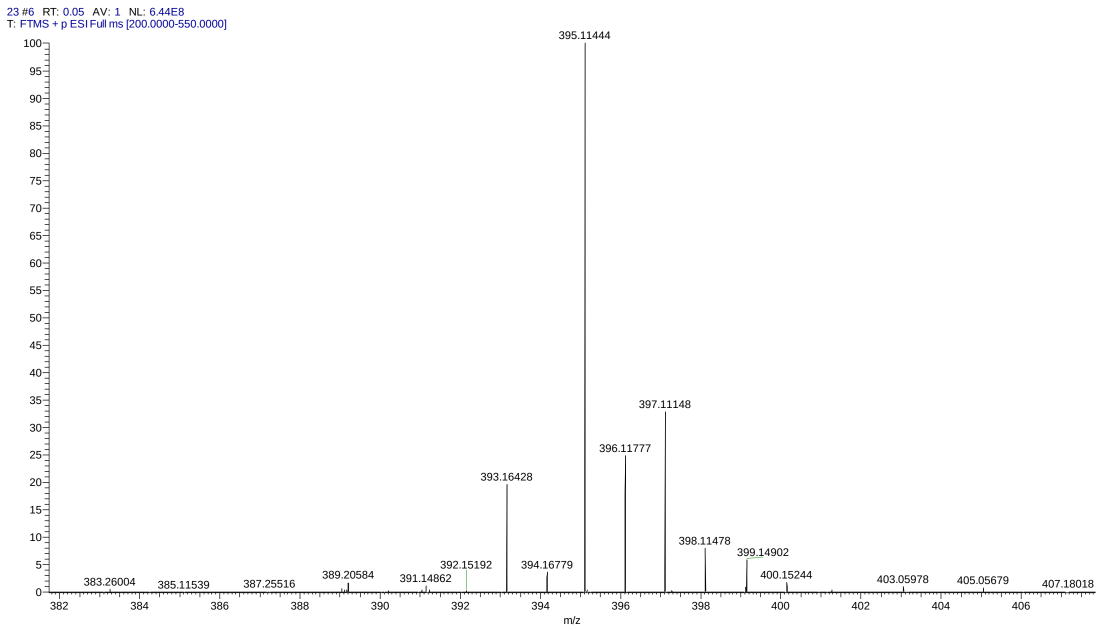

Figure S40. ESI-HRMS spectrum of **11**.

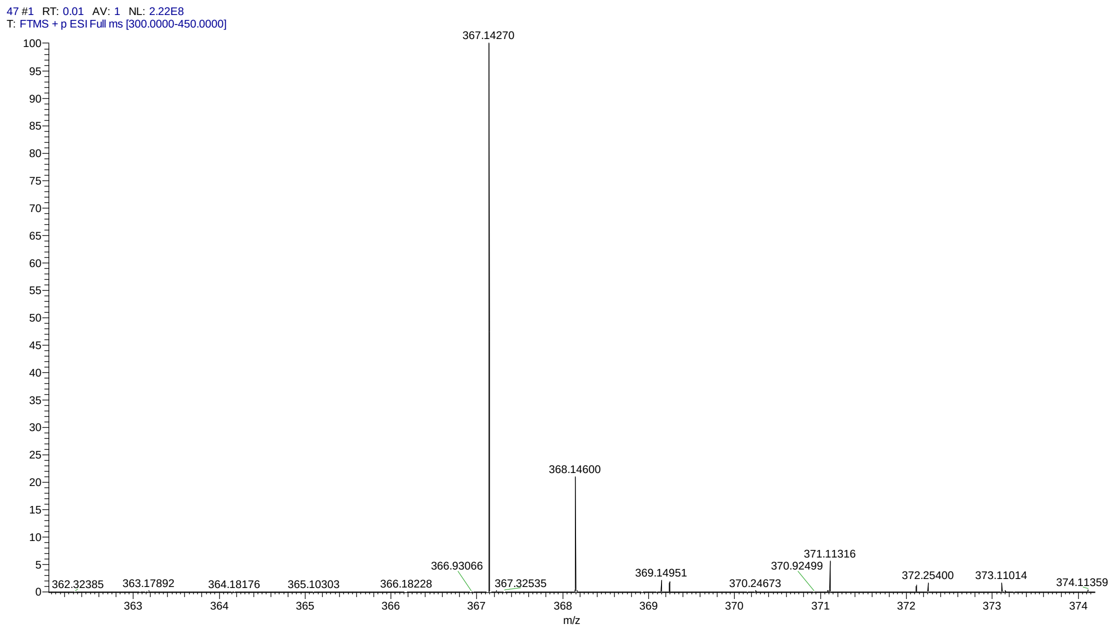

Figure S41. ESI-HRMS spectrum of **12**.

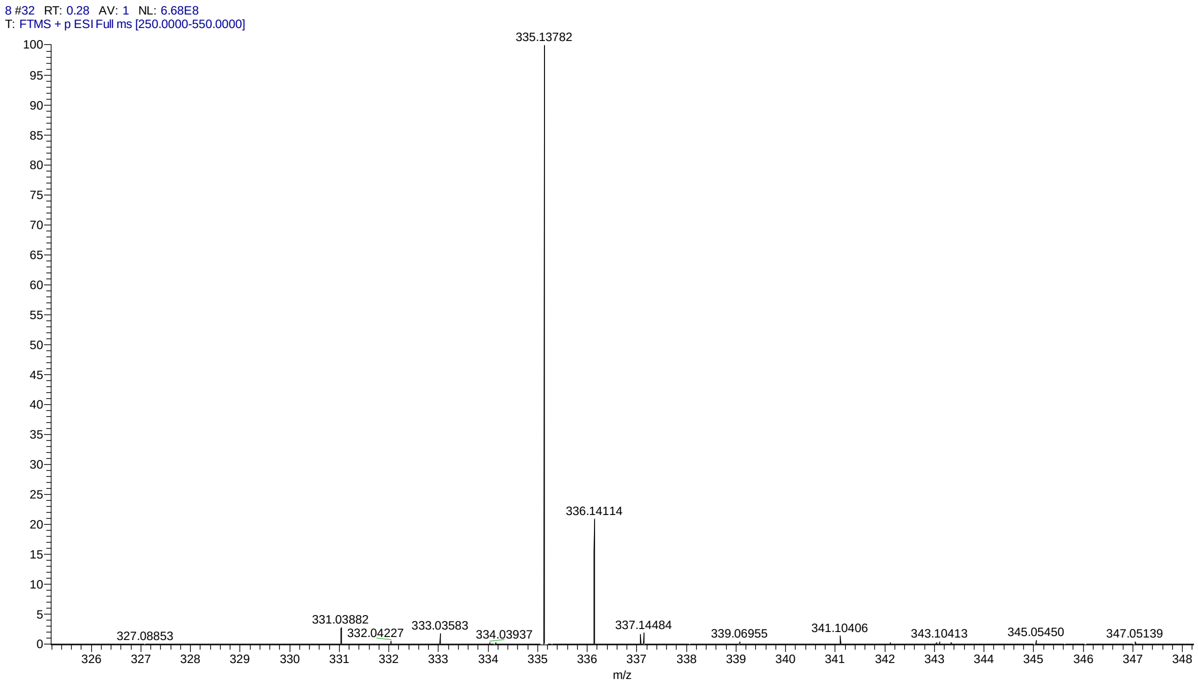

Figure S42. ESI-HRMS spectrum of **13**.

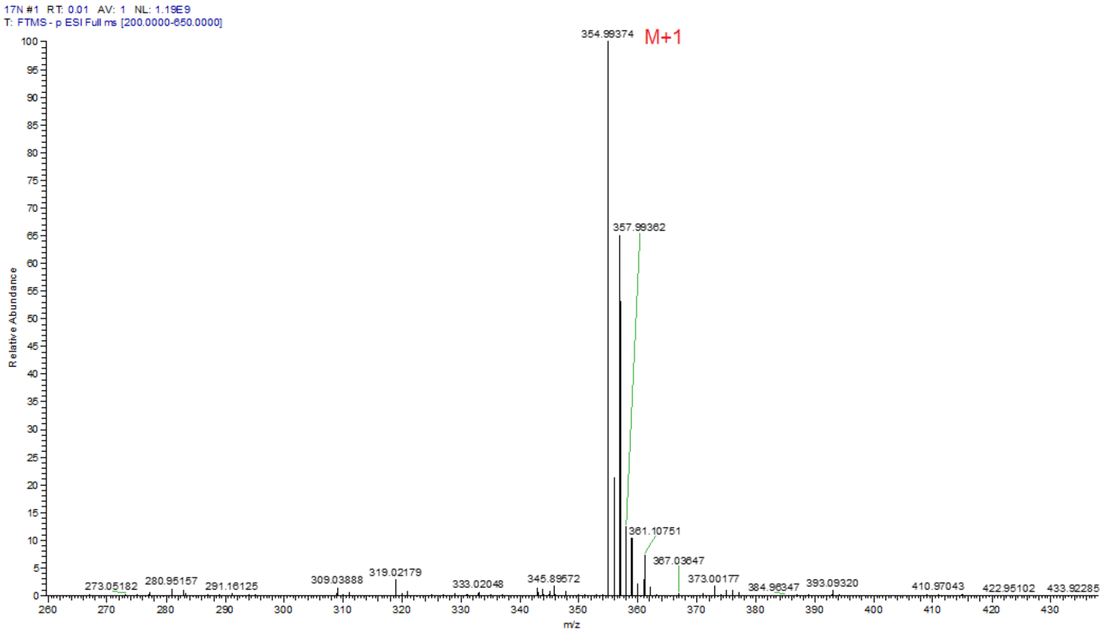

Figure S43. ESI-HRMS spectrum of **14**.

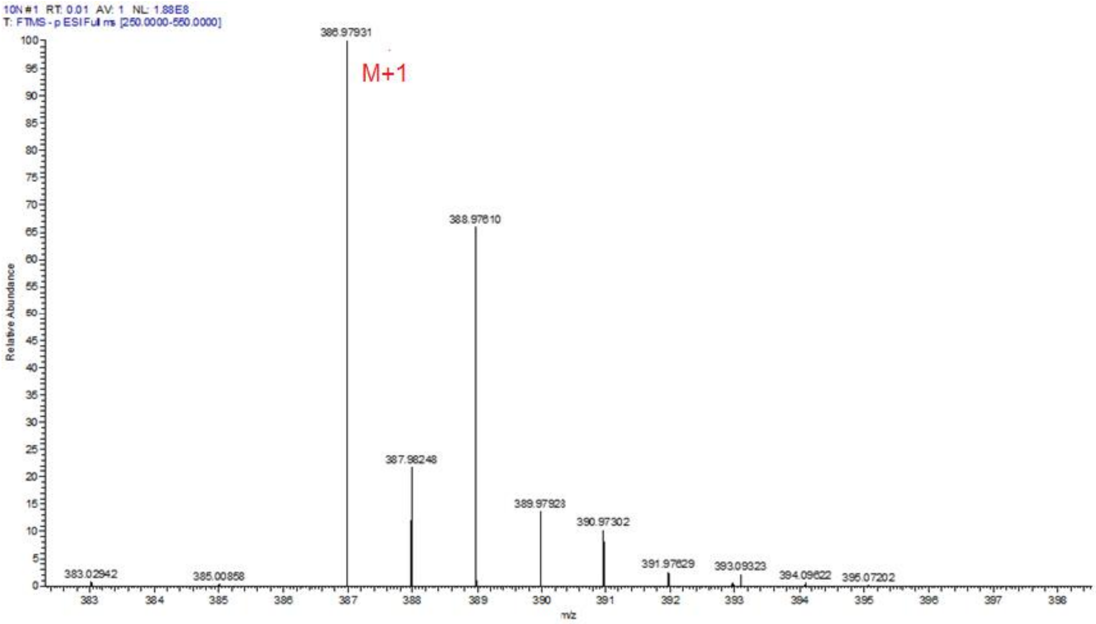

Figure S44. ESI-HRMS spectrum of **15**.

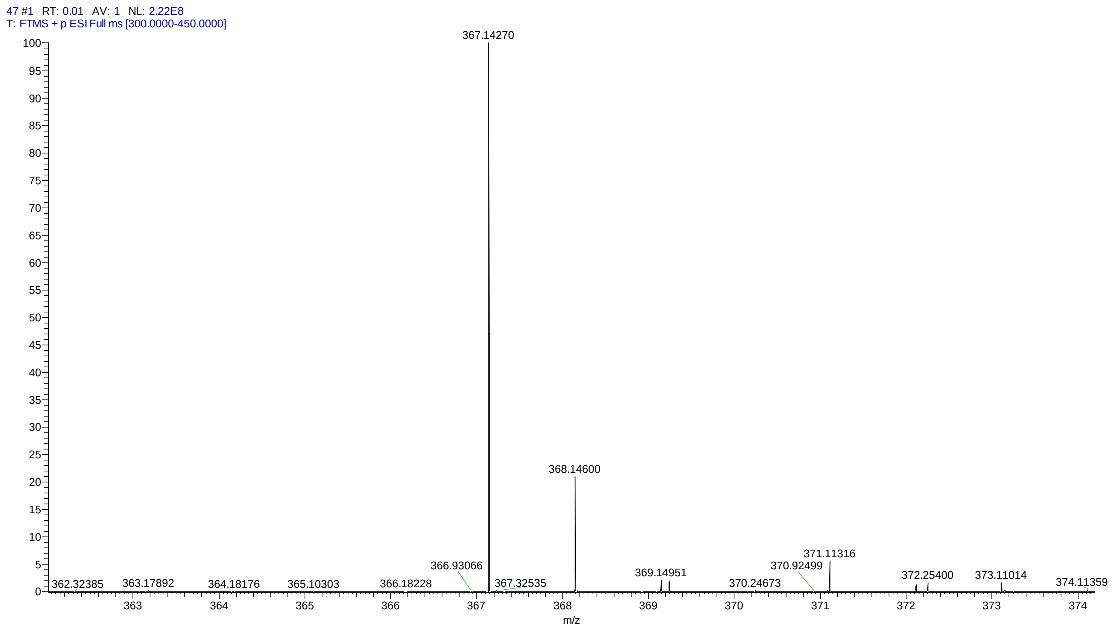

Figure S45. ESI-HRMS spectrum of **16.**

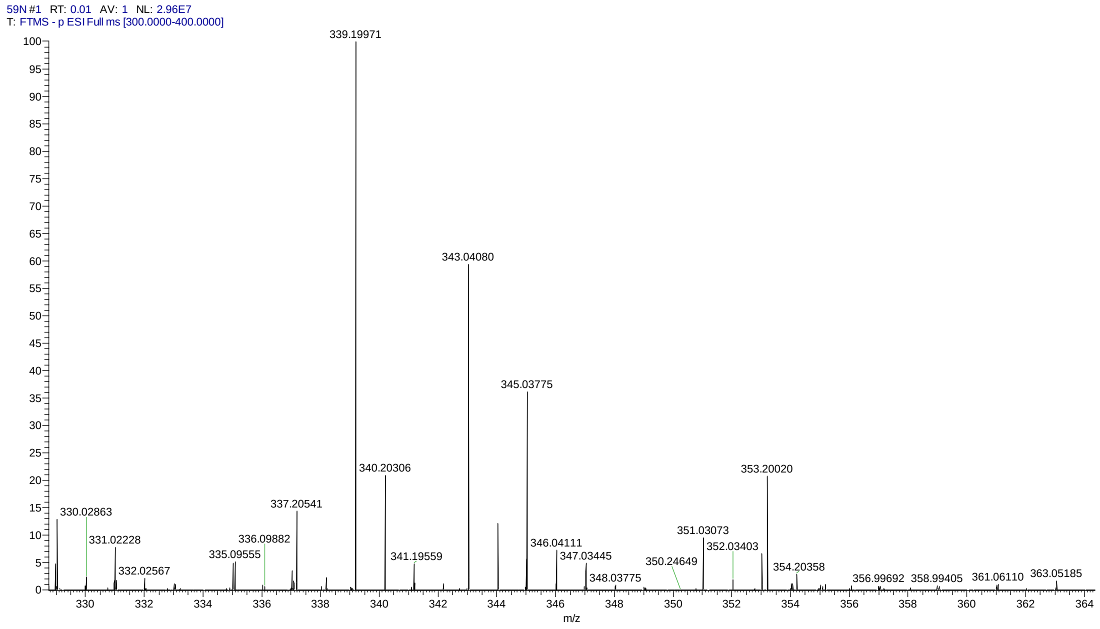

Figure S46. ESI-HRMS spectrum of **17.**

Table S1. The calculated quantum chemical parameters of compounds (**3-17**) using B3LYP/6-31++G(d,p) Level.

Compound	Calculated quantum chemical parameters											
	E_{HOMO}	E_{LUMO}	I	A	ΔE	η	μ	χ	Pİ	ω	ε	dipol	Energy
**3**	-6.0815	-1.7611	6.0815	1.7611	4.3204	2.1602	0.4629	3.9213	-3.9213	3.5591	0.2810	4.0265	-23940.7790
**4**	-6.2279	-1.9331	6.2279	1.9331	4.2948	2.1474	0.4657	4.0805	-4.0805	3.8769	0.2579	2.3375	-35377.9017
**5**	-6.1653	-1.8509	6.1653	1.8509	4.3144	2.1572	0.4636	4.0081	-4.0081	3.7236	0.2686	5.1226	-47883.8499
**6**	-6.2247	-3.0082	6.2247	3.0082	3.2164	1.6082	0.6218	4.6164	-4.6164	6.6259	0.1509	1.9031	-28434.3096
**7**	-6.1996	-1.8975	6.1996	1.8975	4.3022	2.1511	0.4649	4.0485	-4.0485	3.8099	0.2625	2.4353	-25571.8061
**8**	-6.1471	-1.9592	6.1471	1.9592	4.1879	2.0939	0.4776	4.0532	-4.0532	3.9228	0.2549	6.0609	-30229.7363
**9**	-6.2810	-2.1225	6.2810	2.1225	4.1585	2.0792	0.4809	4.2017	-4.2017	4.2455	0.2355	3.1428	-41666.8590
**10**	-6.2149	-2.0414	6.2149	2.0414	4.1734	2.0867	0.4792	4.1281	-4.1281	4.0833	0.2449	7.1348	-54172.8117
**11**	-8.7796	-0.8052	8.7796	0.8052	7.9744	3.9872	0.2508	4.7924	-4.7924	2.8801	0.3472	9.6628	-34506.2911
**12**	-6.2554	-2.0893	6.2554	2.0893	4.1661	2.0830	0.4801	4.1724	-4.1724	4.1786	0.2393	3.5844	-31860.7614
**13**	-6.0633	-1.7750	6.0633	1.7750	4.2883	2.1441	0.4664	3.9192	-3.9192	3.5818	0.2792	3.1695	-30138.5236
**14**	-6.2026	-1.9386	6.2026	1.9386	4.2641	2.1320	0.4690	4.0706	-4.0706	3.8859	0.2573	2.6026	-41575.6525
**15**	-6.2026	-1.9386	6.2026	1.9386	4.2641	2.1320	0.4690	4.0706	-4.0706	3.8859	0.2573	2.6026	-41575.6525
**16**	-6.2021	-2.9895	6.2021	2.9895	3.2126	1.6063	0.6225	4.5958	-4.5958	6.5744	0.1521	3.2850	-34632.0574
**17**	-6.1687	1.9045	6.1687	-1.9045	8.0732	4.0366	0.2477	2.1321	-2.1321	0.5631	1.7760	2.4411	-31769.5544

Table S2. Numerical values of the docking parameters of compounds (**3-17)** against enzymes (kcal/mol).

**1JIJ**	**Docking Score**	**Glide ligand efficiency**	**Glide hbond**	**Glide evdw**	**Glide ecoul**	**Glide emodel**	**Glide energy**	**Glide einternal**	**Glide posenum**
3	-6.67	-0.32	-0.16	-43.53	-2.32	-65.18	-45.85	0.41	237
4	-6.46	-0.31	-0.16	-41.77	-3.56	-63.55	-45.33	1.47	342
5	-6.94	-0.32	-0.16	-46.71	-4.61	-72.42	-51.32	2.38	13
6	-7.14	-0.31	-0.15	-48.29	-5.09	-75.44	-53.39	3.65	119
7	-7.12	-0.34	-0.16	-43.07	-1.87	-65.11	-44.94	1.23	351
8	-6.18	-0.24	-0.16	-46.49	-3.54	-67.62	-50.03	5.53	285
9	-6.35	-0.24	-0.16	-47.67	-4.57	-71.18	-52.24	5.82	95
10	-6.16	-0.23	0.00	-47.35	-4.69	-67.87	-52.04	4.11	292
11	-5.88	-0.21	0.00	-49.51	-4.74	-70.84	-54.25	4.92	396
12	-6.83	-0.26	0.00	-46.75	-3.50	-70.52	-50.26	4.28	358
13	-7.31	-0.25	-0.20	-45.05	-3.71	-68.49	-48.77	5.12	1
14	-6.56	-0.26	-0.20	-45.15	-5.20	-73.16	-50.35	4.71	40
15	-5.64	-0.22	-0.16	-45.73	3.68	-57.45	-42.04	3.25	340
16	-6.83	-0.25	-0.06	-47.30	-4.76	-72.67	-52.05	7.60	259
17	-6.55	-0.26	-0.20	-43.22	-5.22	-69.99	-48.44	4.88	365
Amikacin	-6.07	-0.15	-0.36	-28.77	-23.49	-78.46	-52.27	10.46	372
**2UV0**	**Docking Score**	**Glide ligand efficiency**	**Glide hbond**	**Glide evdw**	**Glide ecoul**	**Glide emodel**	**Glide energy**	**Glide einternal**	**Glide posenum**
3	-3.97	-0.19	-0.11	-21.14	-4.46	-32.06	-25.60	0.78	359
4	-3.82	-0.18	0.00	-22.76	-4.40	-32.91	-27.15	0.65	195
5	-2.83	-0.13	0.00	-23.09	-1.15	-29.02	-24.24	0.31	256
6	-3.92	-0.17	-0.32	-22.49	-5.54	-34.81	-28.03	1.47	263
7	-4.52	-0.22	0.00	-25.49	-0.40	-32.81	-25.88	0.30	214
8	-3.70	-0.14	-0.12	-25.59	-5.65	-36.82	-31.24	2.28	246
9	-3.89	-0.15	-0.30	-28.21	-2.44	-37.49	-30.65	1.56	30
10	-2.88	-0.11	-0.15	-21.05	-8.59	-33.87	-29.65	3.68	147
11	-5.34	-0.19	-0.32	-29.48	-8.16	-47.37	-37.64	4.19	6
12	-4.44	-0.17	-0.40	-27.27	-7.20	-41.57	-34.47	2.26	102
13	-5.69	-0.11	0.00	-19.45	-5.00	-29.44	-24.45	2.70	157
14	-3.28	-0.13	-0.20	-22.92	-3.26	-32.63	-26.18	1.51	87

15	-2.42	-0.09	-0.20	-18.43	-6.00	-28.85	-24.43	2.26	379
16	-2.98	-0.11	-0.16	-20.02	-5.85	-32.51	-25.88	1.29	158
17	-3.05	-0.12	-0.37	-20.88	-1.95	-28.51	-22.83	1.33	4
Amikacin	-4.13	-0.10	0.00	-22.71	-30.13	-65.36	-52.84	21.50	252
**4WUB**	**Docking Score**	**Glide ligand efficiency**	**Glide hbond**	**Glide evdw**	**Glide ecoul**	**Glide emodel**	**Glide energy**	**Glide einternal**	**Glide posenum**
3	-5.11	-0.24	-0.03	-31.64	-1.72	-42.57	-33.36	2.07	326
4	-4.61	-0.22	-0.02	-32.09	-0.22	-40.26	-32.32	2.25	279
5	-5.15	-0.23	-0.02	-29.07	-3.54	-41.93	-32.61	3.00	210
6	-5.65	-0.25	-0.09	-29.58	-5.47	-46.40	-35.04	2.21	337
7	-5.74	-0.24	-0.04	-30.15	-0.69	-39.21	-30.83	1.72	357
8	-5.26	-0.20	0.00	-39.40	-2.37	-51.93	-41.77	7.30	213
9	-4.82	-0.19	0.00	-39.57	-1.43	-50.19	-41.00	6.62	318
10	-4.62	-0.17	0.00	-35.07	-2.53	-47.04	-37.59	2.47	335
11	-5.72	-0.20	0.00	-38.35	-5.52	-56.67	-43.87	6.71	296
12	-5.10	-0.20	0.00	-37.62	-1.44	-48.53	-39.06	6.67	98
13	-4.65	-0.19	-0.10	-28.96	-1.29	-37.10	-30.25	3.39	49
14	-4.12	-0.16	-0.15	-30.59	-0.87	-37.67	-31.46	4.14	35
15	-3.02	-0.12	-0.20	-23.14	-2.72	-31.43	-25.86	1.72	133
16	-3.89	-0.14	-0.16	-29.41	-2.26	-38.51	-31.68	1.72	346
17	-4.68	-0.19	0.00	-34.52	0.79	-39.55	-33.73	6.31	194
Amikacin	-5.07	-0.13	-0.20	-16.41	-29.44	-70.26	-45.86	6.21	362

Table S3. ADME properties of compounds (**3-17**).

	**3**	**4**	**5**	**6**	**7**	**8**	**9**	**10**	**11**	**12**	**13**	**14**	**15**	**16**	**17**	**Reference Range**
mol_MW	276	297	331	307	280	346	367	401	377	350	334	355	389	365	338	130-725
dipole (D)	5.7	3.3	6.9	9.9	3.4	9.7	6.9	10.9	11.2	7.0	8.0	5.2	9.2	10.3	5.3	1.0-12.5
SASA	562	555	557	558	539	655	648	649	651	633	626	619	619	622	603	300-1000
FOSA	123	35	24	26	36	251	164	151	154	164	159	72	59	63	72	0-750
FISA	113	113	111	195	113	96	96	95	179	96	150	150	149	233	150	7-330
PISA	327	335	315	336	344	309	317	299	319	327	317	325	306	326	334	0-450
WPSA	0	72	106	0	47	0	72	105	0	47	0	72	105	0	47	0-175
volume (A^3)	962	948	971	968	918	1175	1161	1185	1182	1132	1096	1082	1106	1103	1053	500-2000
donorHB	1	1	1	1	1	0	0	0	0	0	1	1	1	1	1	0-6
accptHB	2	2	2	3	2	4	4	4	5	4	4	4	4	5	4	2.0-20.0
glob (Sphere =1)	0.8	0.8	0.9	0.8	0.8	0.8	0.8	0.8	0.8	0.8	0.8	0.8	0.8	0.8	0.8	0.75-0.95
QPpolrz (A^3)	33.5	33.1	33.8	33.2	31.9	40.0	39.5	40.3	39.7	38.4	37.5	37.0	37.8	37.3	36.0	13.0-70.0
QPlogPC16	10.4	10.9	11.2	11.2	9.8	12.0	12.4	12.7	12.7	11.4	11.9	12.3	12.7	12.6	11.3	4.0-18.0
QPlogPoct	14.0	13.9	14.9	15.8	13.4	16.3	16.0	17.2	17.5	15.5	16.9	16.6	17.8	18.3	16.2	8.0-35.0
QPlogPw	6.8	6.9	6.8	8.2	6.9	6.7	6.7	6.6	8.0	6.7	8.7	8.8	8.6	10.1	8.8	4.0-45.0
QPlogPo/w	4.1	4.3	4.5	3.1	4.0	4.7	4.9	5.2	3.7	4.6	4.3	4.5	4.8	3.4	4.3	-2.0-6.5
QPlogS	-5.3	-5.5	-5.7	-4.7	-5.1	-5.6	-5.7	-5.9	-4.8	-5.3	-5.4	-5.6	-5.7	-4.8	-5.2	-6.5-0.5
CIQPlogS	-5.1	-5.5	-6.2	-5.3	-5.1	-5.7	-6.1	-6.8	-5.8	-5.7	-5.5	-5.9	-6.6	-5.7	-5.6	-6.5-0.5
QPlogHERG	-5.7	-5.7	-5.4	-5.6	-5.6	-5.9	-5.9	-5.6	-5.8	-5.8	-4.1	-4.1	-3.9	-4.0	-4.1	*
QPPCaco (nm/sec)	846	846	879	139	846	1225	1225	1256	201	1225	95	95	97	16	95	**
QPlogBB	-0.7	-0.5	-0.4	-1.5	-0.6	-0.7	-0.6	-0.4	-1.6	-0.6	-1.2	-1.0	-0.9	-2.1	-1.1	-3.0-1.2
QPPMDCK (nm/sec)	413	1024	1642	58	744	616	1528	2374	87	1111	49	122	190	7	89	**
QPlogKp	-2.2	-2.1	-2.2	-3.6	-2.1	-1.6	-1.6	-1.6	-3.0	-1.6	-2.7	-2.7	-2.7	-4.1	-2.6	Kp in cm/hr
IP (ev)	9.6	9.7	9.7	10.0	9.8	9.8	9.8	9.9	10.2	10.0	9.7	9.7	9.8	10.1	9.9	7.9-10.5
EA (eV)	1.0	1.1	1.1	2.4	1.2	1.3	1.4	1.4	2.5	1.5	1.2	1.3	1.3	2.5	1.4	-0.9-1.7
#metab	2	1	1	2	1	4	3	3	4	3	3	2	2	3	2	1-8
QPlogKhsa	0.7	0.7	0.7	0.5	0.6	0.7	0.7	0.7	0.4	0.6	0.4	0.4	0.5	0.2	0.3	-1.5-1.5
Human Oral Absorption	3	3	3	3	3	3	3	3	3	3	3	3	3	2	3	-
Percent Human Oral Absorption	100	100	100	84	100	100	100	100	90	100	88	89	91	68	87	***

PSA	60	60	60	105	60	65	65	65	110	65	89	89	89	134	89	7-200
RuleOfFive	0	0	0	0	0	0	0	1	0	0	0	0	0	0	0	Maximum is 4
RuleOfThree	0	0	0	0	0	0	1	1	0	0	0	0	1	1	0	Maximum is 3
Jm	0.0	0.0	0.0	0.0	0.0	0.0	0.0	0.0	0.0	0.0	0.0	0.0	0.0	0.0	0.0	-

* corcern below -5, **<25 is poor and >500 is great, *** <25% is poor and >80% is high.
